# Genetic history of the population of Crete

**DOI:** 10.1111/ahg.12328

**Published:** 2019-06-13

**Authors:** Petros Drineas, Fotis Tsetsos, Anna Plantinga, Iosif Lazaridis, Evangelia Yannaki, Anna Razou, Katerina Kanaki, Manolis Michalodimitrakis, Francisco Perez‐Jimenez, Giustina De Silvestro, Maria C. Renda, John A. Stamatoyannopoulos, Kenneth K Kidd, Brian L. Browning, Peristera Paschou, George Stamatoyannopoulos

**Affiliations:** ^1^ Department of Computer Science Purdue University West Lafayette Indiana; ^2^ Department of Molecular Biology and Genetics Democritus University of Thrace Alexandroupolis Greece; ^3^ Department of Biostatistics University of Washington Seattle Washington; ^4^ Department of Genetics Harvard Medical School Boston Massachusetts; ^5^ Department of Hematology George Papanicolaou Hospital Thessaloniki Greece; ^6^ Department of Forensic Medicine University of Crete Heraklion Crete Greece; ^7^ IMIBIC/Reina Sofia University Hospital University of Cordoba Cordoba Spain; ^8^ Transfusion Medicine Department General Hospital‐Padua University Padova Italy; ^9^ Unita di Ricerca P. Cutino Ospedali Riunti Villa Sofia‐Cervello Palermo Italy; ^10^ Departments of Medicine and Genome Sciences University of Washington Seattle Washington; ^11^ Department of Genetics Yale University School of Medicine New Haven Connecticut; ^12^ Department of Medicine, Division of Medical Genetics University of Washington Seattle Washington; ^13^ Department of Biological Sciences Purdue University West Lafayette Indiana

**Keywords:** crete, greece, historical genetics, medieval history, population genetics, whole‐genome

## Abstract

The medieval history of several populations often suffers from scarcity of contemporary records resulting in contradictory and sometimes biased interpretations by historians. This is the situation with the population of the island of Crete, which remained relatively undisturbed until the Middle Ages when multiple wars, invasions, and occupations by foreigners took place. Historians have considered the effects of the occupation of Crete by the Arabs (in the 9th and 10th centuries C.E.) and the Venetians (in the 13th to the 17th centuries C.E.) to the local population. To obtain insights on such effects from a genetic perspective, we studied representative samples from 17 Cretan districts using the Illumina 1 million or 2.5 million arrays and compared the Cretans to the populations of origin of the medieval conquerors and settlers. Highlights of our findings include (1) small genetic contributions from the Arab occupation to the extant Cretan population, (2) low genetic contribution of the Venetians to the extant Cretan population, and (3) evidence of a genetic relationship among the Cretans and Central, Northern, and Eastern Europeans, which could be explained by the settlement in the island of northern origin tribes during the medieval period. Our results show how the interaction between genetics and the historical record can help shed light on the historical record.

## 1 INTRODUCTION

The population history of Crete can be traced to the early Neolithic when the island was colonized by farmers from Anatolia who established in Knossos, at about 7000 B.C.E., one of the first Neolithic settlements in Europe (Evans, 1994); other Neolithic settlements were subsequently established all over Crete (Tomkins, 2008). These Neolithic settlers and subsequent waves of Neolithic migrants (Broodbank & Strasser, 1991; Cherry, 1981; Nowicki, 2008; Weinberg, 1965) established the first advanced European civilization, the Minoan civilization, which flourished in Crete from 3000 to about 1450 B.C.E. (Evans, 1921). The island was subsequently conquered by the Myceneans of mainland Greece (Bennet, 2011; Chadwick, 1976; deFidio, 2008) who ruled from around 1450 to 1100 B.C.E. Homer (1650) describes Crete as a populous island with 90 cities inhabited by several tribes: the Achaeans, who correspond to the people now called Myceneans (Bennet, 2011; Schofield, 2007); the Pelasgians who were the pre‐Hellenic population of the Helladic space (Herodotus, 1999; Strabo, 2006); the Eteocretans (Cretans of the old stock); the Kydonians; and the Dorians (Strabo, 2006). Eteocretans and Kydonians were considered to be autochthonous Cretans while the other tribes originated from Greece (Strabo, 2006). There is little of significance coming from Crete during the classical times other than the frequent wars between the city‐states (Detorakis, 2015). Following the Hellenistic period during which there is no record of population migrations to Crete, the island was conquered in 69 B.C.E. by the Romans (Sanders, 1982). The almost 400 years of Roman occupation was followed by about 500 years of relatively peaceful rule by the Byzantines (Tsougarakis, 1988) until Crete fell in 827 C.E. to Arab exiles from Andalusia (Brooks, 1913; Christides, 1984; Detorakis, 2015; Vassiliev, 1980). The Arab Emirate of Crete was frequently raided the Aegean and Eastern Mediterranean, but after 134 years of Arab rule, the island was recaptured in 961 C.E. by the Byzantines (Norwich, 1998; Vassiliev, 1980). The 243 years of the second Byzantine rule ended when the Byzantine Empire fell to the Francs and the Venetians of the Fourth Crusade. The Venetians purchased the island in 1204 C.E. from the crusader Boniface of Montferrat; they ruled Crete for 465 years and established a feudal system that provoked several revolutions of the population (Detorakis, 2015; Xanthoudidis, 1939). From 1645 to 1669, Ottomans and Venetians fought for 24 years over Crete and the island was captured by the Ottomans who ruled for 267 years during which the Cretans revolted several times (Detorakis, 2015). The island gained its autonomy in 1889 and was unified with Greece in 1913.

Historians have for long debated the effects the medieval historical events may have had on the Cretan population. Ancient written sources are typically concise and noncontemporary, hence theories and interpretations of historians are sometimes based on extrapolations or on a (perhaps subjective) synthesis of inadequate information. Population genetics analysis can complement historical approaches by detecting and quantifying changes in population structure as a result of migrations and population admixture, but, to the best of our knowledge, no Cretan genetic analyses based on whole‐genome autosomal markers have appeared in prior work. A number of studies have been conducted using Y DNA (Di Giacomo et al., 2003; King et al., 2008; Malaspina et al., 2001; Martinez et al., 2007; Voskarides et al., 2016) or mtDNA (Hughey et al., 2013; Martinez, Mirabal, Luis, & Herrera, 2008), generating results that raised interesting points for investigation, but with nondefinite conclusions, limited by the amount of information that a small number of genetic loci can provide. The earliest studies on Y DNA had shown considerable heterogeneity between the Cretans (Di Giacomo et al., 2003; Malaspina et al., 2001), with later studies correlating haplotypes from various areas of Crete with Balkan, Italian, Anatolian, or even paleolithic populations (King et al., 2008; Martinez et al., 2007; Voskarides et al., 2016). The study of mtDNA initially supported the previous findings, especially on the matter of paleolithic signatures, along with a Middle Eastern component in the Cretan population (Martinez et al., 2008). The study of ancient mtDNA from Crete identified high genetic affinity of the ancient Cretans with modern Cretans and Neolithic Europeans (Hughey et al., 2013). Here we ask whether population genetics can help provide insights on the impact of various historical events on the genetic structure of the population of Crete.

## 2 SUBJECTS AND METHODS

### 2.1 Design of the study and geographic districts

**Figure 1:**
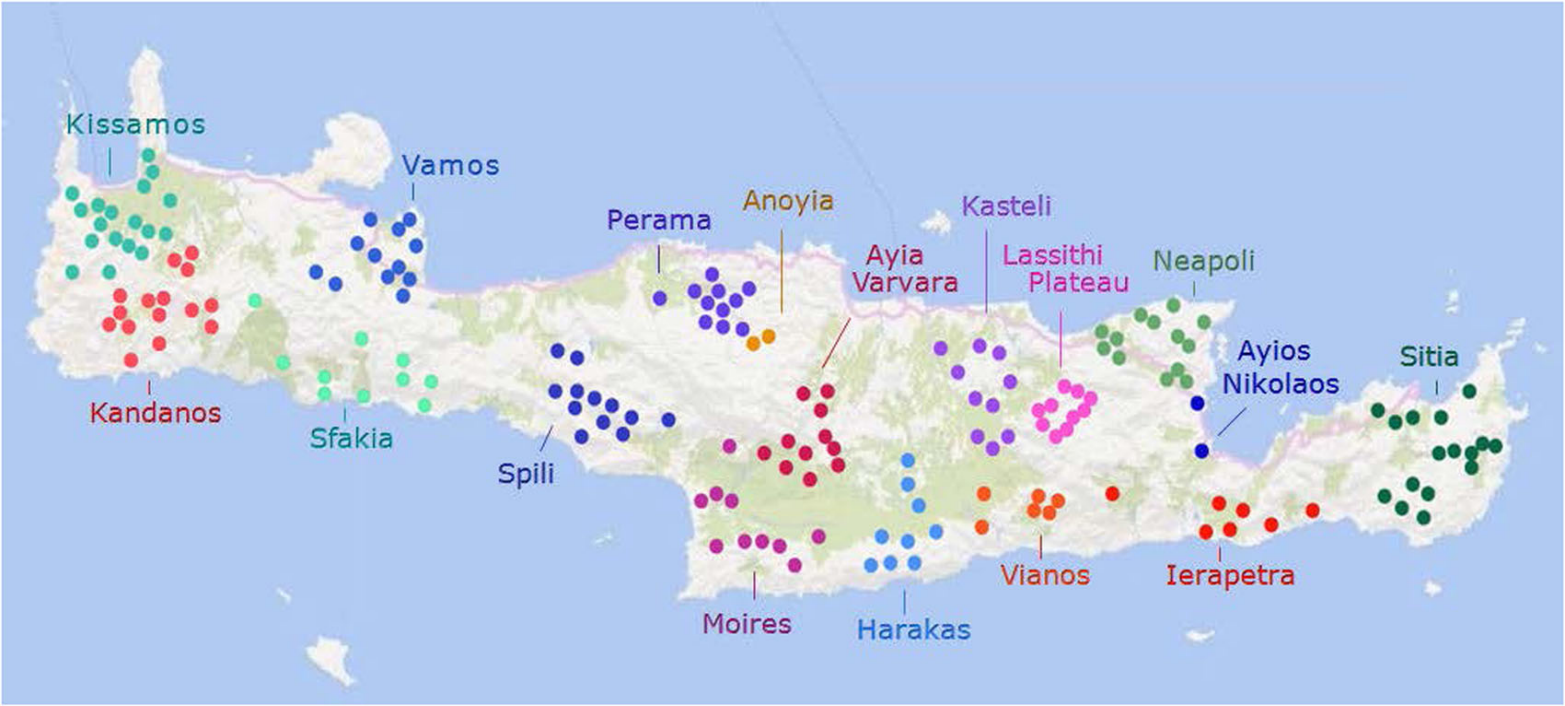
Locations of the origins of the grandparents of the participants of the study. Each of the 17 Cretan subpopulations is shown with a different color. The origins of the grandparents represent fairly well the distribution of the rural population of Crete in the second half of the 19th century [Color figure can be viewed at http://wileyonlinelibrary.com]

The study has been reviewed by the appropriate committees of the University of Washington and the University of Crete. All participants have signed informed consent for using their DNA for molecular analysis including sequencing. We focused on the rural population of Crete. The participants were males or females 70 years or older (range of ages 70 to 94 years) who had paternal and maternal grandparents originating from villages located in the same district of Crete. With this approach, we reconstructed the rural population of Crete at the time of birth of the grandparents of the participants (i.e., the population of the period 1850 to 1880). According to the 1881 census, the Greek population of Crete consisted of 205,000 individuals (Stratakis, 1890), 80% of whom were living in 1,150 villages and hamlets. 131 individuals were included in the study and their grandparents originated from 171 villages (Figure 1).

Crete was divided into 17 districts (Figure 1, Table S1) corresponding to the Health Centers through which health care is provided to the rural population. The four cities (Chania, Rethymno, Irakleio, Sitia) and their suburbs were excluded from the study. The names of the 17 districts in the figures and the supplemental tables correspond to the locations of the Health Centers. The distribution of the Health Centers (and of the associated centers of rural infirmaries in remote villages) agrees well with the distribution of the rural population of Crete. The 171 villages (and two small towns, Ayios Nikolaos and Anoyia) of origin of the study participants are distributed relatively evenly over rural Crete (Figure 1; see supplementary text for a more detailed overview of the Cretan ethnogeography).

### 2.2 Datasets used for the analyses

To explore relationships beyond the regions of the Cretan populations, we analyzed samples from published datasets from populations from around the world (Figure S1). Samples from Greek subpopulations and populations from the area of Cordoba in Andalusia, from Veneto and from Sicily collected in the context of this study, are listed in Table S2. Additionally, the refined identity by descent (IBD) analysis included 185 Greek samples genotyped on the Illumina 2.5M version 1.1 arrays. Table S2 also lists samples from the Human Genome Diversity Project (http://www.hagsc.org/hgdp), the 1000 Genomes Project (http://www.1000genomes.org), and other sources.

### 2.3 Merging genotypes from different sources

To merge datasets from the different sources described in Table S2, we had to pay particular attention to strand information and properly align common single‐nucleotide polymorphisms (SNPs). As such information was not always available, we chose to omit SNPs with alleles C/G and A/T to avoid ambiguity.

### 2.4 Quality control

Despite the fact that the missing genotype rates in any of the datasets that we analyzed in our work were quite low (invariably below 1%), we chose to perform an additional quality control check and remove any SNP that had a missing rate exceeding 20% in any of the populations under study when merging datasets. Our objective was to remove SNPs that might cause spurious artifacts in our analyses simply because they had many missing genotypes in one of the studied populations.

### 2.5 Principal Component Analysis (PCA)

We used Eigenstrat (Price et al., 2006) to perform PCA on the available data (and subsets thereof). A detailed discussion of PCA and its use in population genetics appears in Price et al. (2006) and Paschou et al. (2007a, 2007b).

### 2.6 Estimating population admixture

We used the ADMIXTURE v1.22 software for all our admixture analyses. The parameter *K* (number of ancestral populations) ranged between two and eight in all our analyses. Prior to running ADMIXTURE, we pruned the SNPs to remove SNPs in high linkage disequilibrium (LD). Toward that end, we used the PLINK software and pruned SNPs using a windowed approach and a value of *r* ^2^ equal to 0.8. We used DISTRUCT v.1.1 and CLUMPP v.1.1.2 to visualize the output of ADMIXTURE (Alexander, Novembre, & Lange, 2009). We also used the meta‐analysis technique developed in Stamatoyannopoulos et al. (2017) to perform a meta‐analysis of the ADMIXTURE output.

### 2.7 Identity by descent (IBD)

Two datasets were used in the IBD analyses. The first dataset included 759 samples from Crete, the Peloponnese, Greece, and parts of southern Europe (Sicily, Serbia, Veneto, Tuscany, Andalusia, Basque, Iberia, Italy, and Sardinia), with 619,756 SNPs in common prior to processing. The second dataset included 1,882 samples with 275,180 SNPs in common. A map of the origins of the samples in this second dataset is shown in Figure S1 with a corresponding list of populations in Table S2.

Allele strand, reference, and alternate alleles were aligned with the 1000 Genomes European populations (CEU, GBR, TSI, FIN, and IBS), using the conform‐gt Beagle Utility (http://faculty.washington.edu/browning/conform-gt.html) prior to running Beagle. 566 and 590 markers, respectively, were excluded because of an inability to confirm the strand orientation. Of the remaining markers, any with minor allele frequency less than 1%, Hardy–Weinberg *P*‐values less than 10^−6^, or containing more than 2% missing data were excluded. Thus 560,891 SNPs were included for the dataset with 759 individuals, and 257,945 SNPs were included for the dataset with 1,882 individuals.

We used the Refined IBD algorithm implemented in Beagle 4.1 (Browning & Browning, 2013) to phase the data and infer IBD segments. Default values were used for all of the parameters except that we set niterations to 160. Per the software recommendations, the ibdtrim parameter was set to the average number of markers in 120 kb, which was 22 for the dataset with 749 individuals and 10 for the dataset with 1,882 individuals. We used the HapMap genetic map. We excluded IBD segments with length <2 cM, as Refined IBD has been shown to have a low false‐positive rate when using this threshold (Browning & Browning, 2013). We required a logarithm‐of‐odds (LOD) score of 3 when inferring IBD segments, which means that the probability of the observed genotype data for a pair of samples in the inferred IBD segment is at least 1,000 times greater under an IBD model than under a non‐IBD model.

R Core Team (2014; http://www.R-project.org) was used to generate plots and summarize IBD distributions. The heat maps summarize average pairwise IBD between chromosomes from different individuals in the populations being compared. Specifically, we calculated the sum of the lengths of IBD segments shared between a pair of individuals in the two populations (or within a population), then divided by the number of unique pairs of individuals in the two populations (or within a population) and by four times the genome length (for the four ways to pair chromosomes between two people). We excluded pairs of individuals with more than 40% IBD sharing. This threshold excludes half siblings, full siblings, and avuncular pairs.

For the bootstrap analyses, we sampled individuals with replacement from each of the populations being compared, up to the original sample size of that population. We then recalculated the average IBD sharing as described above. We repeated this procedure for 500 bootstrap samples and calculated the mean and 95% confidence interval for the average pairwise IBD between the populations.

### 2.8 Network analysis

To better understand the connection between populations included in our study, we performed a network analysis on the results of PCA, following the lines of our prior work (Paschou et al., 2014). To form the networks, we identified the top few nearest neighbors of each sample by representing each sample with respect to the top *K* coefficients returned by PCA and then computing the distance of each sample to all other samples, under the additional constraint that these neighbors should not belong to the same population of origin as the sample itself. Once a network whose nodes correspond to populations and whose edges correspond to connections between populations, as described above, is formed, we visualize it using the Cytoscape software package (see Paschou et al., 2014 for details).

### 2.9 ChromoPainter and FineSTRUCTURE

To further investigate the connections between Cretan populations, as well as their connections to their Southern European neighbors, we used the ChromoPainter and FineSTRUCTURE (Lawson, Hellenthal, Myers, & Falush, 2012) pipeline to analyze population structure within Crete as well as a combined dataset including Cretan data and Southern European populations. More specifically, our first dataset in this analysis included all Cretan populations, while our second dataset in this analysis included Sardinia, Sicily, Italy, Tuscany, Veneto, Basque, Andalusian, Iberia, and all Cretan samples. For visualization purposes only, the Cretan samples were split in eastern Crete, central Crete, and western Crete, based on geography. ChromoPainter and FineSTRUCTURE are methods that use inferred haplotypes to depict haplotypic sharing through a chromosome painting method. We used SHAPEIT (Delaneau, Marchini, & Zagury, 2012) to infer haplotypes from genome‐wide markers on the studied populations. The resulting haplotypes were then used as input to the ChromoPainter and FineSTRUCTURE pipeline to achieve a detailed representation of the shared haplotypic chunks. ChromoPainter utilizes a method of haplotypic painting that depicts the shared haplotypic segments between individuals given a shared ancestral donor via a Hidden Markov Model. It calculates the effective population size, the mutation rate, and the effective number of chunks. FineSTRUCTURE was used in two independent Markov Chain Monte Carlo runs, using 100,000 total iterations (50,000 for the Markov Chain Monte Carlo burn‐in step and 50,000 for the actual random walk). The painted chromosomes are combined using the chromo‐combine step. The derived co‐ancestry matrix is then used for a hierarchical clustering tree based on the Markov Chain Monte Carlo step. We plotted the results using a combination of Python plotting scripts and the native R scripts provided by the authors.

## 3 RESULTS

### 3.1 Genetic characterization of the Cretan populations

The Cretan population sample consisted of 129 individuals originating from 17 rural districts (Figure 1; Table S1). The subjects were at least 70 years old with all four grandparents originating from villages of the same rural district. The subjects were genotyped with the Illumina 1M or 2.5M arrays and compared with the populations listed in Table S2.

**Figure 2:**
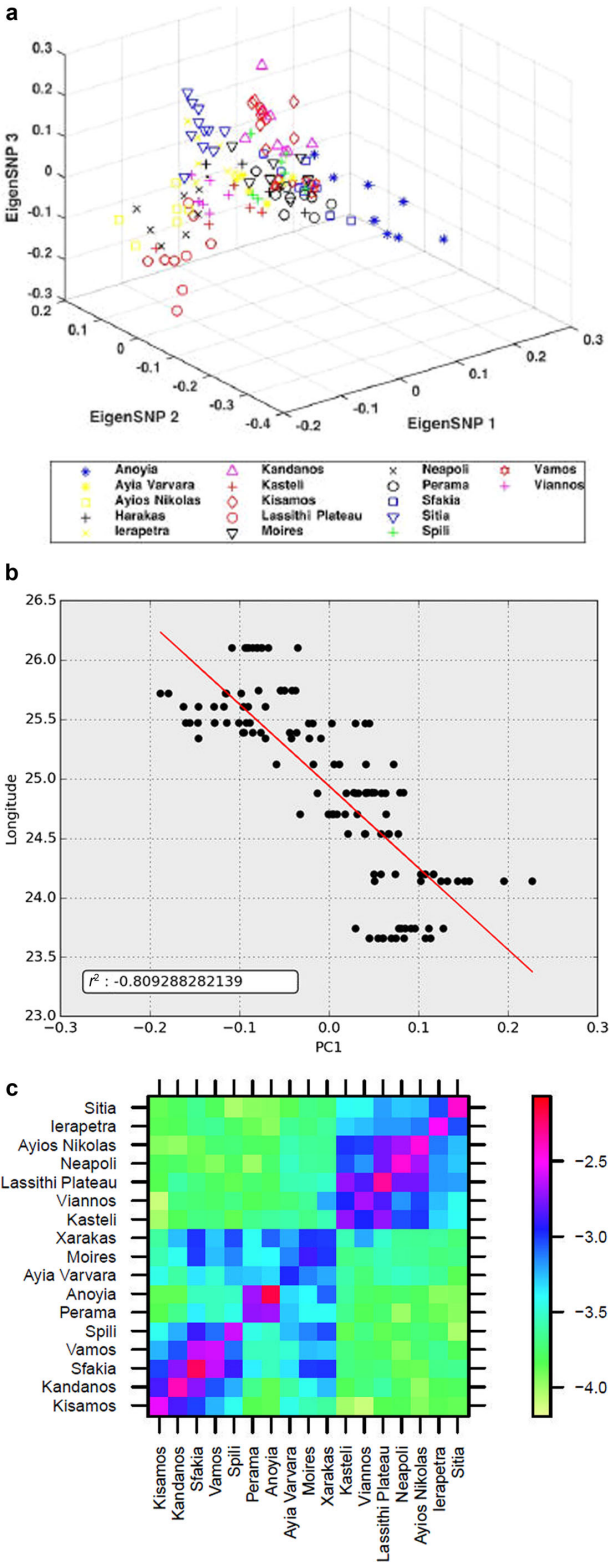
Genetic differentiation of the Cretan populations. (a) Results of principal component analysis (PCA) analysis of the 17 Cretan subpopulations. Notice that the individuals of the study are not distributed randomly but they form clusters distinguishing several subpopulations. Also notice that the eastern and the western subpopulations are placed on the opposite sites of the graph. Anoyia, Lassithi, Sitia, Sfakia, and Kisamos are clearly separated in the top three principal components. Some overlap exists between the remaining populations. (b) Correlation between geographic coordinates (longitude) and the top principal component. Notice that the east‐to‐west axis of the Cretan island is captured extremely well by the top principal component demonstrating the east‐to‐west gradient in gene frequencies. There is essentially no correlation between latitude and the second top component (*r* ^2^ is approximately 0.15). (c) Heat map of the log of the average proportion of genome shared identity by descent (IBD) between a pair of individuals in the specified districts of Crete. Higher values (less negative; toward the red end of the color scale) indicate higher IBD sharing. The populations are placed sequentially according to their location on the island from the east to the west. Notice that they form three clusters: an eastern, a central, and a western. Noticeable also in the central cluster is a subcluster formed by the populations of Anoyia and Perama [Color figure can be viewed at http://wileyonlinelibrary.com]

Several subpopulations known for their distinct cultural characteristics and contributions to the Cretan history can be discerned when we observe the results of the PCA analysis. The population of Sfakia in western Crete, with a history of multiple revolutions against conquerors (Detorakis, 2015), shows a clustering of its individuals in Figures 2a and S2. Lassithi plateau in Mount Dicte is a geographic isolate inhabited continuously since the Neolithic; it has several ancient defense sites (Nowicki, 2000) and served as a refugium at times of upheaval throughout the Cretan history. Some clustering of individuals of the populations of Lassithi Plateau can be seen in Figures 2a and S2. Other subpopulations that are at least partially separated by PCA are Anoyia and Perama (Figures 2a and S2) in the northern slopes of Mount Ida, the Eastern‐most population of Sitia (Figure S2) and the Western‐most populations of Kissamos and Kandanos (Figure S2). Several of these subpopulations could also be (at least partially) distinguished by ADMIXTURE (Figure S3). The findings of ChromoPainter and FineSTRUCTURE are broadly in agreement with PCA and ADMIXTURE analyses, as shown in the dendrogram of Figure S3b. Indeed, in the dendrogram, populations are clustered mostly based on relative latitude and there is no co‐clustering of populations at the two latitudinal ends of Crete.

Another feature of the Cretan genetic diversity is an east‐to‐west gradient in gene frequencies shown in the PCA plot of Figure S2a and in the correlation between longitude and the top principal component of Figure 2b. A pronounced east‐to‐west gradient is also apparent in the ADMIXTURE analysis of Figure S3a (*K* = 2, *K* = 3) and in the IBD sharing heat‐plot of Figure 2c. This is in contrast to the north‐to‐south axis, which is not captured well by PCA (Figure S4). This is not surprising given the geography of the Cretan island and how narrow the north‐to‐south axis of the island is.

The east‐to‐west gradient could represent ancient population settlement patterns. It is known that the Minoan settlements concentrated in central and eastern Crete (Branigan, 1970) while the Myceneans (likely of Peloponnesean origin) dominated the central and the western parts of the island (deFidio, 2008). The Kydonians inhabited western Crete and the Eteocretans inhabited southern (Strabo, 2006) and eastern Crete (Duhoux, 1982). Eastern Crete received waves of new immigrants from the Anatolian coast through the Dodecanese in the Final Neolithic/Early Minoan (around 3,500 to 3,000 B.C.E.) (Nowicki, 2002, 2008). It was thus possible that the east‐to‐west gradient reflected these old population distributions that had been preserved by the geography of the island. Compatible with population movements between Crete, Peloponnese, and Dodecanese are the findings of IBD analysis (Figure S5) showing high IBD sharing between Peloponnese and west Crete and similarly high between Dodecanese and east Crete. Another explanation of the east‐to‐the west gradient, supported by the Cretan mountainous geography, is isolation by distance. Future analyses might help clarify this issue.

**Figure 3:**
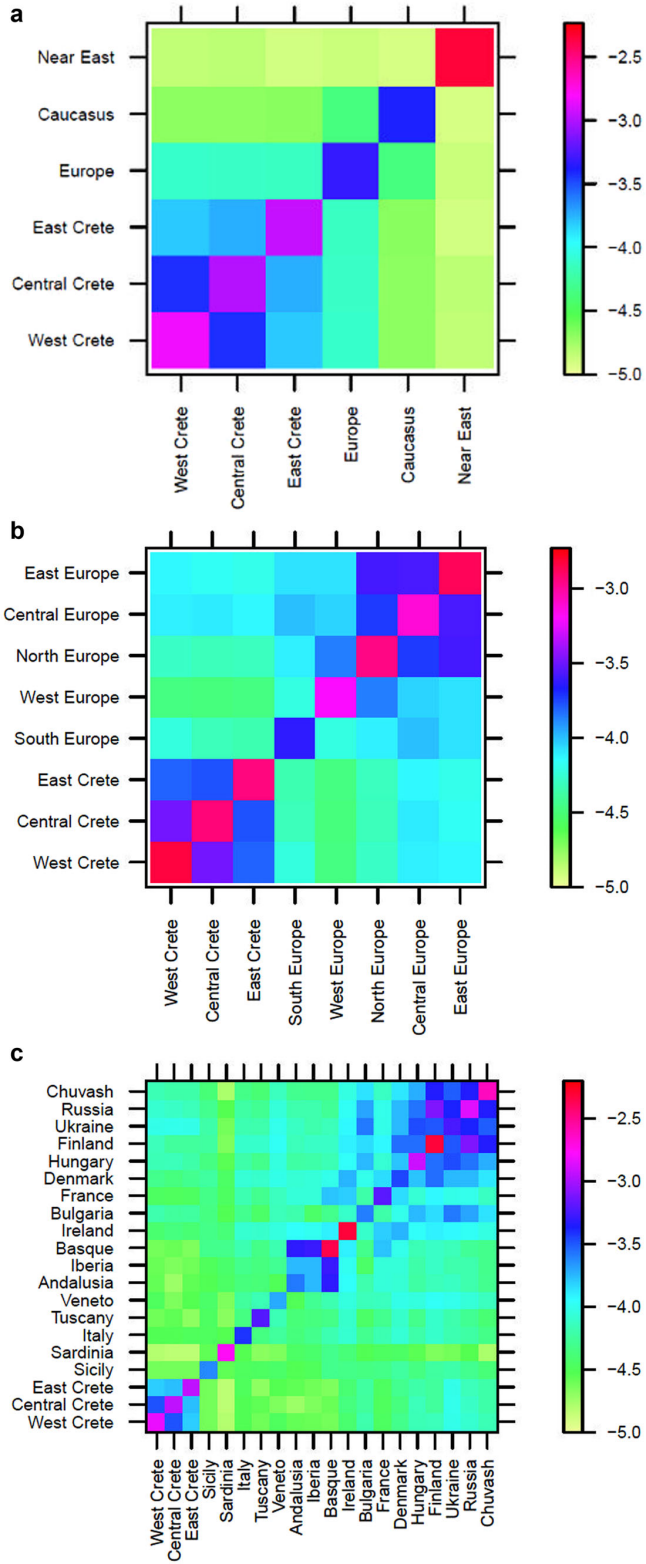
Heat maps of the log of the average proportion of genome shared identity by descent (IBD) between a pair of individuals in the specified populations. Higher values (less negative; toward the red end of the color scale) indicate higher IBD sharing. (a) Relationships among Crete, Europe, the Caucasus, and the Near East. Europe includes Belarusians, Estonians, Germans, Lithuanians, Orcadians, Poles, and Swedes. Caucasus includes Georgians, Armenians, Abhkasians, and the Adygei. The Near East includes Bedouins, Druze, Jordanians, Palestinians, Samaritans, and Syrians. (b) Relationships between Crete and the Eastern, Central, Northern and Western regions of Europe. Eastern Europe includes Chuvash and Russia. Central Europe includes Hungary and Ukraine. South Europe includes Sicily, Peloponnese, Serbia, Veneto, Tuscany, Andalusia, Basque, Iberia, Italy, and Sardinia. Northern Europe includes Denmark, Finland, and Ireland. Western Europe includes just France. (c) Relationships between Crete and specified European populations. Notice that Crete shares higher IBD relationships with the Eastern and Central European populations [Color figure can be viewed at http://wileyonlinelibrary.com]

To explore the genetic relationships between Cretans and the European and Near Eastern populations, we employed IBD analysis and PCA. In IBD analysis, our primary measure of relatedness was mean pairwise IBD, that is, the average amount of detected IBD (in segments >2 cM) shared between individuals in two populations (Tables S3 and S4). The heat map in Figure 3a shows the average amount of IBD shared between individuals in Crete, Europe, the Caucasus, and the Near East. All three regions of Crete are most strongly related to Europe. Bootstrap analyses confirm that the Crete–Europe relationship is significantly stronger than either Crete–Caucasus or Crete–Near East (Figure S6).

Within Europe, Crete is most closely related to Central and Eastern Europe (Figure 3b and 3c; Tables S3 and S4). However, the difference between Southern, Central, and Eastern Europe is borderline statistically significant (Figure S7). Among the non‐Greek populations of Table S4, east Crete shares the highest IBD with Ukraine (proportion of pairs with IBD 41.6% and mean pairwise IBD 1.39 cM) and Poland (proportion of pairs with IBD 39.5% and mean pairwise IBD 1.29 cM). West Crete (Table S3) shares the highest proportion of pairs with IBD and mean pairwise IBD with Poland (42% and 1.48 cM) and Ukraine (39.9% and 1.37 cM). None of the Southern European populations share high levels of IBD with Crete (Figures 3c and S8). However, most differences in mean pairwise IBD between Crete and individual European populations are not statistically significant (Figures S9 and S10).

**Figure 4:**
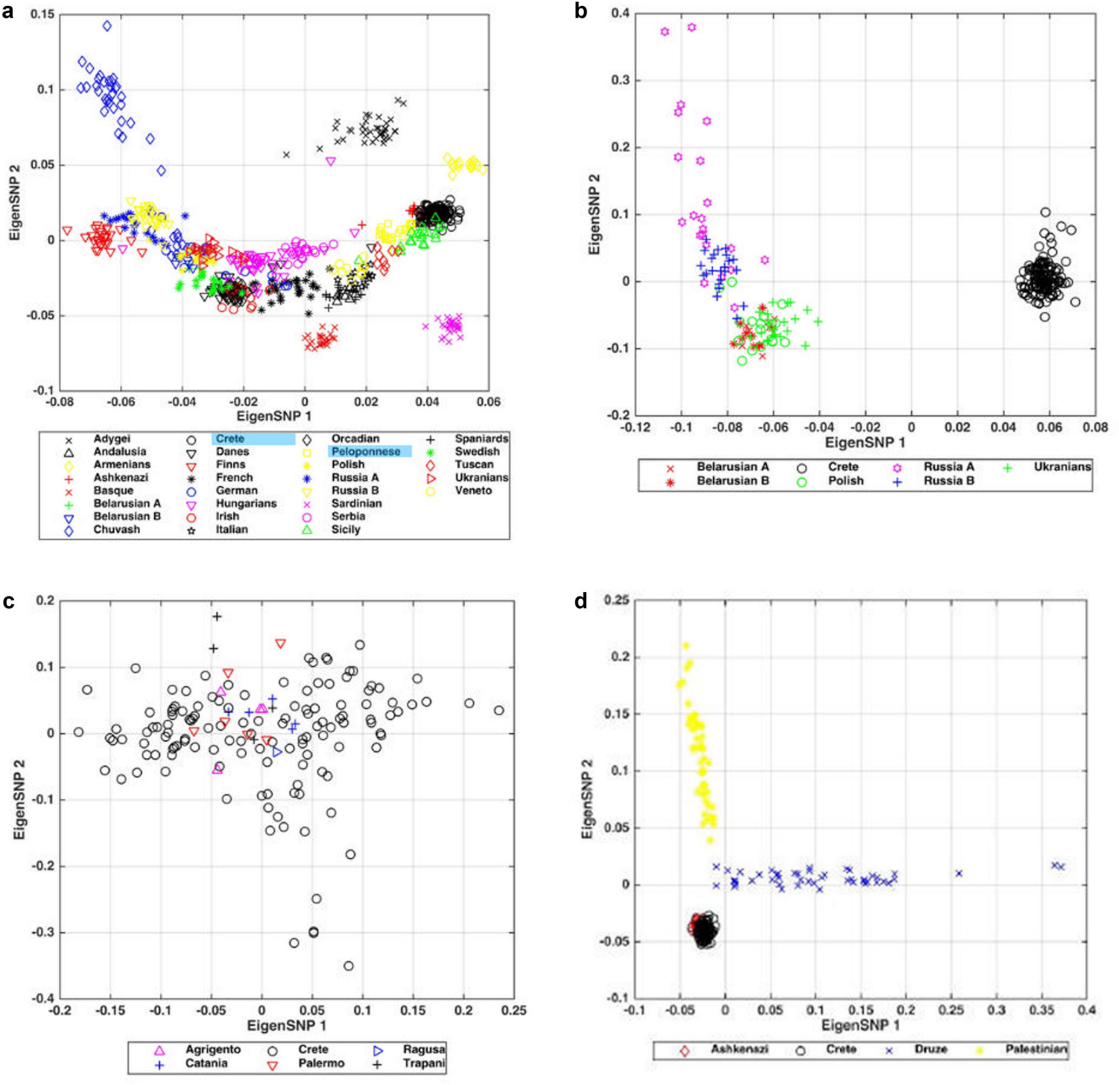
Principal component analysis (PCA) plots for the Cretan and European populations. The first two principal components are shown in all plots. (a) Cretans and European populations: there is a clear north‐to‐south cline along the first eigenvector, with the north appearing on the left side and the south on the right side of the plot. Highlighted in blue are the labels of the Greek populations of Crete and Peloponnese. The second eigenvector captures some of the east‐to‐west cline and also shows the genetic distance of the Cretans from the Armenians and the Adygei, two populations from the region of Caucasus. The Sardinians and the Basque are located outside the European cluster in the first eigenvector, while the Chuvash are outside the cluster in the second eigenvector. The Cretans are located near the rightmost part of the plot, overlapping with the Peloponnesian, the Sicilian, and the Ashkenazi populations. (b) PCA results for the Cretan and the Slavic populations. The heterogeneity of the Slavs dominates the plot. The first eigenvector captures the genetic distance between the Cretans and the Slavs. The second eigenvector shows the difference between the Slavic populations, with Russians being the most diverse population along the eigenvector. (c) PCA results for Cretans and Sicily. There is a complete overlap among many of the Sicilian and the Cretan samples. (d) PCA results for Cretans, Ashkenazi, Druze, and Palestinians. When compared by PCA to the Palestinian and the Druze, Cretans and Ashkenazi overlap and they both separate from these Near Eastern Semitic populations [Color figure can be viewed at http://wileyonlinelibrary.com]

In contrast to the IBD data, PCA comparisons of Crete with the European populations distinguish the Cretans from Central, Northern, and Eastern Europeans (Figures 4a and S11a). PCA plots specifically show a clear separation of the Cretans from the Polish, Ukrainians, Russians, and Belarussians (Figures 4b and S11b). They are also clearly distinguished from Western and Northwestern Europe (Figures 4a and S11b). ADMIXTURE plots confirm the PCA findings (Figures S12 and S13).

The differences in the results of PCA and IBD analysis probably reflect differences in time frames. The genome‐average coalescent time for pairs of samples determines the locations of samples in PCA space (McVean, 2009). Coalescent time at loci with discordant alleles can be very old. In contrast IBD analysis detects recent coalescent events, with the approximate time frame determined by the minimum length threshold for detected IBD segments. Ralph and Coop (2013) estimate that almost all IBD segments >2 cM length in Europeans date from a common ancestor within the past 4,000 years, and most of this sharing derives from common ancestors 1,500–2,500 years ago.

To determine the time frame when the IBD relationships between Crete and Eastern and Central Europe were developed, we applied ALDER (http://groups.csail.mit.edu/cb/alder), a method that uses the decay of admixture LD to estimate the time of a single pulse of admixture and quantify its proportion, using the Cretan population as the mixed population and varying the source population. In Table S5, we list the source populations that provided the strongest evidence of admixture (exponential amplitude and decay more than four standard errors higher than zero). A series of populations from western (CEU), northern (CEU, Estonian), and Eastern (Ukrainian, Russian) Europe produce admixture estimates of approximately 17%–28% dating to the medieval period.

Both PCA and ADMIXTURE point to the strong genetic similarities between Cretans and Southern Europeans, especially the Sicilians (Figures 4a, 4c, and S11c). This genetic relationship is also demonstrated by network analysis of the European populations (Figure S14). Sicily has also been previously placed next to Crete using phylogenetic trees and tree mix analysis (Paschou et al., 2014). These results might reflect the common genetic history of Crete and Sicily rather than gene flow between the two islands. Sicily was heavily colonized by Greeks starting in the eight century BC (Freeman, 1891; Thucydides, 1986). Dorian Greeks colonized the South and the Southeast coast of Sicily while the Ionian Greeks colonized the North and Northeast coast. Sicily continued to be Hellenized in medieval times but under the Norman domination the usage of the Greek language was discouraged and it was eventually replaced by Italian. Southern Italy was also colonized by Ionian, Achaean, and Dorian Greeks, and these colonies, together with Sicily, composed the Greek‐dominated part of Italy, which the Latin speakers called Magna Graecia (Burry, 1963; Ceserani, 2012). The PCA (Figure 4a and 4c) and ADMIXTURE (Figure S15) data show that the historic bonds between Greece (including Crete) and Sicily were not simply cultural but genetic as well. The above findings were broadly confirmed by our ChromoPainter/FineSTRUCTURE analysis (Figure S16a and S16b).

In the PCA of Crete vs Europe, the Cretans overlap with three populations: the Peloponneseans, the Sicilians and the Ashkenazi Jews (see Figures 4a, S17, and S18). Southern European and Mediterranean ancestry of the Ashkenazi Jews has also been demonstrated before (Atzmon et al., 2010; Behar et al., 2010; Bauchet et al., 2007; Price et al., 2008; Seldin et al., 2006; Tian et al., 2008). Furthermore, we find in both PCA and ADMIXTURE analysis, that the Ashkenazi are more similar to the Cretans than to the two Levantine Semitic populations. One possible explanation is that this relation might reveal a common Mediterranean ancestry that the Cretan and Ashkenazi populations share.

### 3.2 Genetic effects of medieval historic events

Three medieval events had major impact on Crete: the conquest of the island by the Andalusian Arabs; the violent re‐capture of Crete by the Byzantines; and the four and half centuries of rule and colonization by the Venetians. To obtain insights we compared the Cretans with the extant populations of origins of the conquerors and settlers assuming that these populations represent reasonably well the populations who settled in Crete during medieval times.

#### 3.2.1 The genetic effects of the Arab occupation

Religious strife and a failed revolution in Arabic Andalusia in the beginning of the 9^th^ century resulted in the exile of a large segment of the population of the area of Cordoba (Dozy, 1913). About 15,000 exiles settled in Morocco (Dozy, 1913; Levi‐Provencal, 1938; Ostrogorsky, 1969) and 12,000, in 827 CE, conquered Crete, established there a piratical state and ruled the island for 134 years (Christides, 1984; Ostrogorsky, 1969; Vassiliev, 1980). Historians refer to that period of Cretan history as “Arab occupation” although the majority of the conquerors were indigenous Andalusians whose ancestors had converted to Islam (Christides, 1984; Dozy, 1913); the term “Arab occupation” will also be used here. The Andalusians were joined by North African Arabs (Cannard, 1986; Dozy, 1913; McVean, 2009) and Moslems from the Near East settled in the island during the 134 years of the Arab rule. To obtain insights on the effects of the Arab occupation we compared the Cretans with Andalusians, North African and Near Eastern populations.

**Figure 5:**
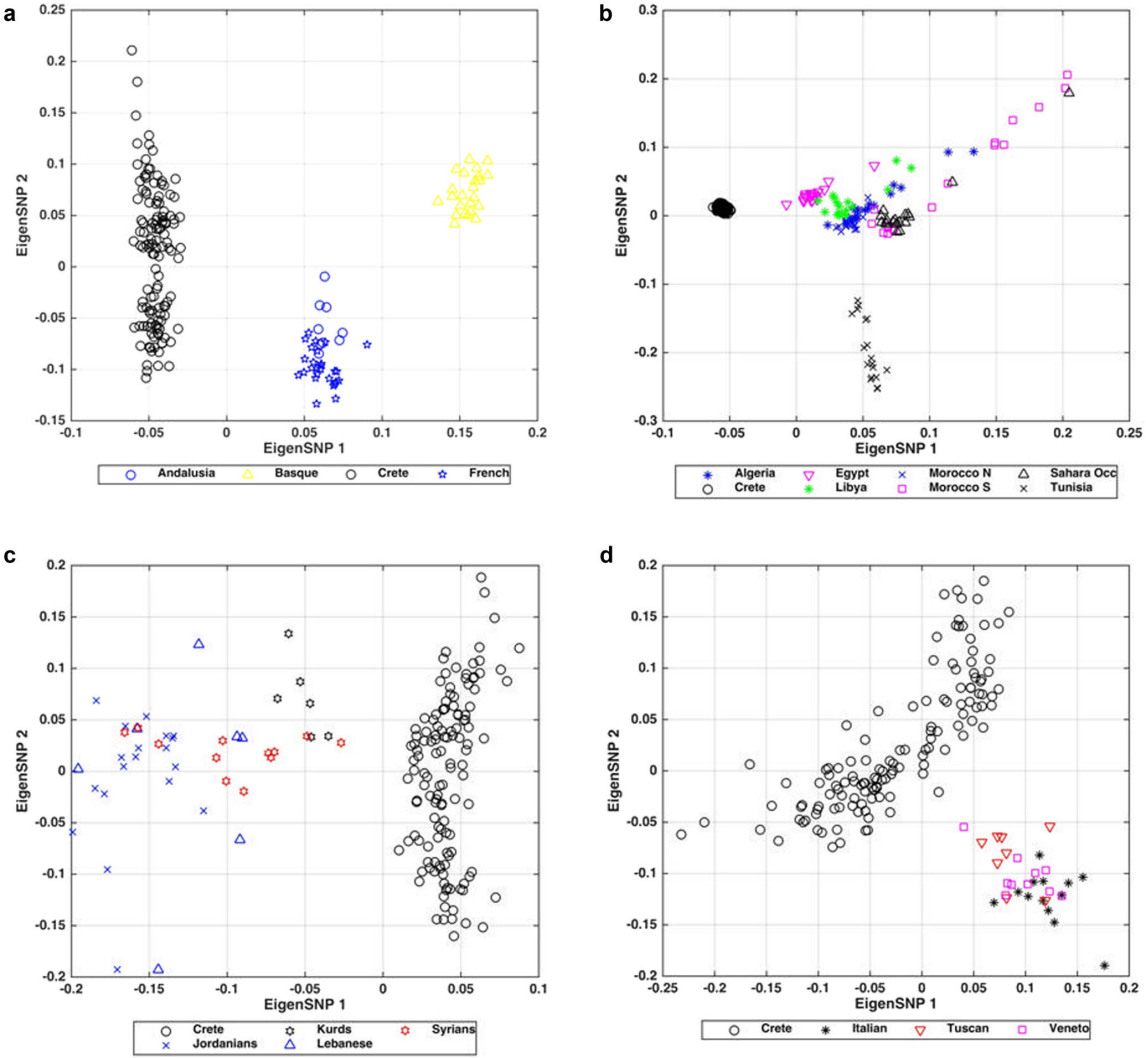
Results of principal component analysis (PCA) comparing Cretans with the populations of origin of the conquerors and settlers of Crete during the medieval period. The top two principal components are shown in all plots. (a) The Cretans are compared with Andalusians who conquered and settled in Crete in the 10th century. The top eigenvector shows a clear distinction between the Cretans, and the Andalusians. Basque and French are also included in the figure. (b) North Africans, especially Tunisians and Moroccans, were prevalent among the Andalusian Arabs who conquered Crete. Notice that the Cretans are clearly distinguished from these North African populations. (c) Medieval Arab sources mention that Crete received settlers from Syria during the Arab occupation. The top eigenvector differentiates the Cretans from the Near Eastern populations. Closer, but clearly separated from the Cretans, are the Kurds and the Syrians. (d) Comparison of the Cretans with the Venetians who occupied and settled Crete for four and a half centuries. Both the first and the second eigenvector separate the Cretans from the Venetians [Color figure can be viewed at http://wileyonlinelibrary.com]

**Figure 6:**
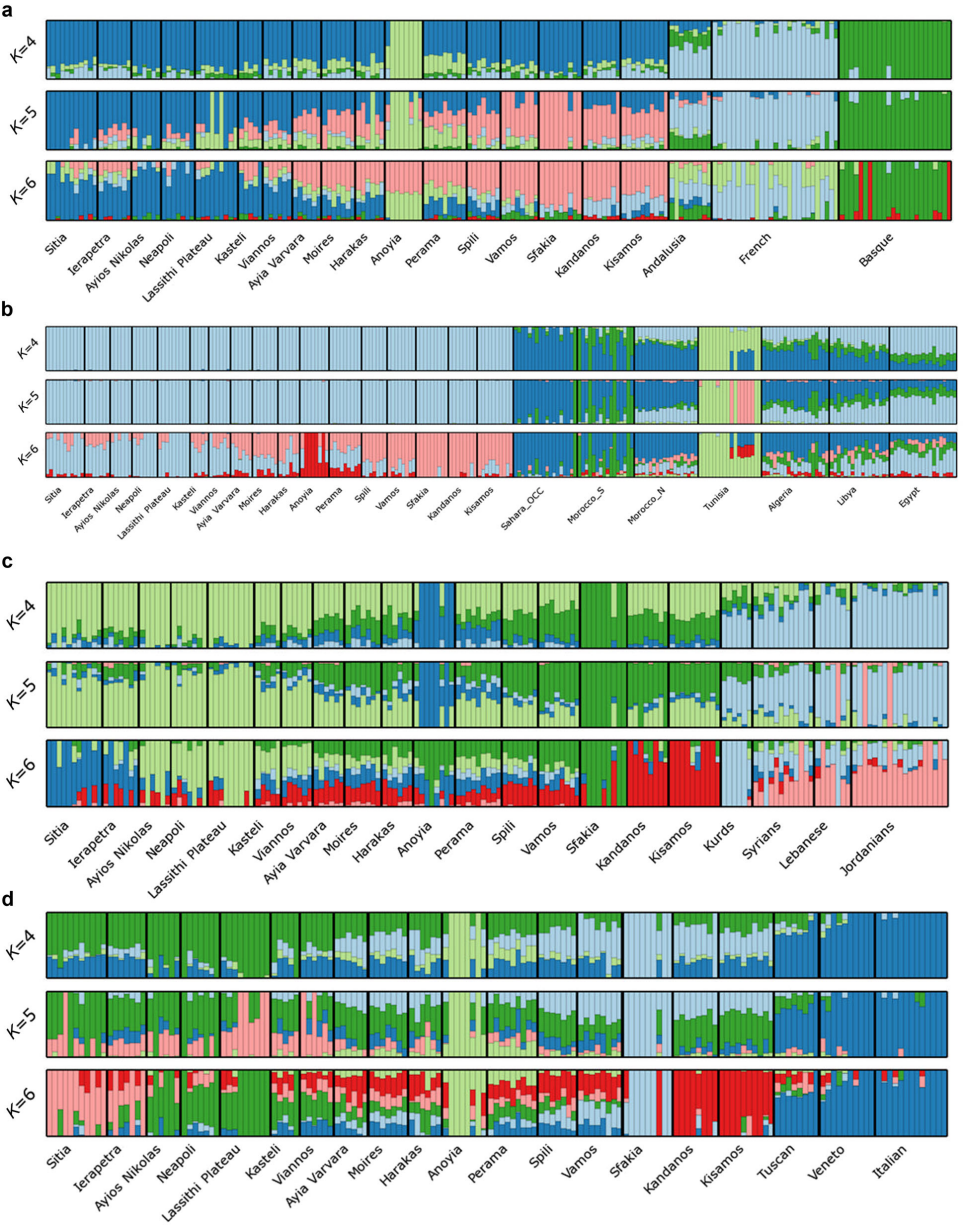
ADMIXTURE analysis for the Cretans and the Andalusians, North Africans, Near Eastern populations, and the Venetians. Results are shown for *K* equal to four, five, and six hypothetical ancestral populations. (a) A possible shared component can be observed between the Andalusian and the Cretans, especially the western populations (Kissamos, Kandanos), especially for *K* equal to six. (b) The North African populations are genetically distinct from the Cretans, except for a small amount of shared ancestry between Tunisians and the Anoyia subpopulation for *K* equal to six. (c) A possible shared component can be observed between the Near East to the Cretans for *K* equal to four or five. (d) Cretans and the Venetian populations appear completely distinct with only very minor shared ancestry. We can also observe a possible shared component between the Italian populations and the Cretan subpopulations [Color figure can be viewed at http://wileyonlinelibrary.com]

PCA (Figures 5a and S19a) clearly separated the Cretan from the Andalusian sample. ADMIXTURE (Figures 6a and S20) further supports the PCA findings. Cretans are also clearly separated by North Africans and Near Eastern populations as shown both by PCA and ADMIXTURE (Figures 5b, S19b, 6b, and S21). Figures 6c and S22 show a gradient from the Cretans toward the Kurds, the Syrians, the Lebanese, and the Jordanians (this is especially striking for *K* = 2)._ Overall, the PCA and ADMIXTURE analyses did not provide evidence that the Arab occupation of Crete resulted in the levels of admixture that would have been recognized by our techniques.

#### 3.2.2 Population settlements by the Byzantines

The 134 years of the Arab rule ended when the army of the Byzantine general Nicephorus Phokas conquered Crete after a year‐long hard‐fought campaign (Panayotakis, 1960; Talbot & Sullivan, 2005). The Byzantines slaughtered or enslaved the Andalusians and the Arabs and perhaps many converts to Islam. The Arab fourteen century historian Nuwayri estimates that 200,000 Moslems perished in Crete (Gaspar, 1904), but this number is considered an exaggeration (Christides, 1984). The extinction of Andalusians, Arabs and Moslems should have created a population void in the island. There is a five words‐long sentence in the book of Leo the Deacon (Talbot & Sullivan, 2005), the contemporary historian who recorded the re‐capture of Crete by the Byzantines, which indicates that the Byzantines tried to fill this void with new settlers. Leo writes that after the extermination of the Arabs, Nicephorus Phokas, settled in Crete “Romans and Armenians and men from other tribes” (Talbot & Sullivan, 2005). The meaning of this statement has been debated; does it refer to the settlement of veterans from the army of Nicephorus Phokas, an event of minor population significance? Or, it refers to settlement to Crete of Byzantine populations from other areas of the Empire, an event that could have had a major impact on the structure of the Cretan population. Re‐settlements of large population groups for political and strategic reasons have been frequently practiced by the Byzantine administration (Charanis, 1961). By “Romans” Leo refers to all the populations of the Byzantine Empire which at that period extended from the Balkans to Syria; we can only speculate what the population origin of these “Romans and other tribes” would have been. Settlements of Armenians most likely occurred and have left traces recognized today in the names of villages (Tomadakis, 1939). Comparison of Cretans and Armenians by PCA and ADMIXTURE showed a clear separation of the two populations (Figures S23 and S24). Armenians shared with the Cretans the lowest proportion of pairs with IBD and mean pairwise IBD (0.20 cM) of all populations of Tables S3 and S4. We conclude that there is no evidence for significant Armenian ancestry in the Cretan rural population.

#### 3.2.3 The genetic effects of the venetian occupation

The Venetians ruled Crete for 435 years, built cities, settled colonists, admixed with the locals and contributed to the creation of a characteristic Cretan cultural renaissance. We compared the Cretans with Italian populations and a population sample from the area of Veneto which includes Venice and the surrounding areas. Figures 5d and S19d show a gradient from the Cretan populations, on the left side of the plot, toward Tuscans, Venetians, and Italians at the right side of the plot. ADMIXTURE analyses (Figures 6d and S25) support the PCA findings; as shown in Figure S25, even for *K* = 2, the gradient from the Cretans to the Tuscans, the Venetians, and eventually the Italians is quite pronounced.

## 4 DISCUSSION

Historians strive hard to reconstruct the history of populations on the basis of often limited sources. Problems are magnified when critical events have not been documented by contemporary writers but they have reached historians by chronologically removed sources. In such cases, historians attempt to assess the impact of events on the basis of perhaps inadequate information and, in doing so, they might be influenced by factors such as political philosophy, ethnicity, etc. This explains the fact that there often exist different interpretations of the same event by different historians. Population genetics can provide relatively unbiased insights on the population effects of historical events (assuming appropriate sample collection and extensive statistical analyses). We attempted to provide such insights regarding the effects of various historical events on the genetics of the population of Crete.

Historians have attempted to analyze the population effects of the Arab occupation of Crete on the basis of a relatively inadequate written record. Except for the campaign of the Byzantine general Nicephorus Phocas to Crete (which has been recorded in detail), little written record exists on the 134 years of Arab occupation. It has been suggested that the Arab occupation had a dramatic effect on the composition of the Cretan population (Sefakas, 1939; Treadgold, 1988). During the Arab rule, the Christian population of the island was largely converted to Islam while Arabs settled in Crete and Cretans sought refuge in lands under the control of the Byzantines. Arab sources have claimed that the whole Christian population of the island was expelled and replaced by Muslims. Comments in Byzantine sources (Bekkerus, 1840; Ceserani, 2012; Panayotakis, 1960; Talbot & Sullivan, 2005) point to population changes but they might refer to changes in religion rather than ethnicity. Opposing views have been presented by historians who claim that the very scant archaeological record left behind by the Arabs (Miles, 1964), the very small number of Arabic toponyms in the island (Detorakis, 2015; Tomadakis, 1960–1961), and the lack of Arabic additions to the Cretan dialect are all evidence of a rather limited impact of Arab occupation on the population of Crete (Christides, 1984; Tomadakis, 1960–1961).

A similar controversy exists on the impact of the 435 years of the Venetian rule. The Venetians were accomplished bureaucrats and left a good record of their administration of the colony. According to those sources, approximately 2,000 feudalists initially colonized Crete in the 13th century. However, many other Venetian and Italian merchants, soldiers, and artisans settled in the urban centers (McKee, 2000) later. Even though marriages between Venetians and Cretans were initially discouraged, there is considerable evidence for admixture between Latins and Cretans (McKee, 1993, 2000; Maltezou, 1995). The cultural and physical admixture was so extensive that even the notion of Greek ethnicity of the Cretans of that era has been challenged (McKee, 2000). Other historians, however, have argued that in spite of the extensive cultural impact, the influence of the Venetians was mostly on the populations of the major cities (Gasparis, 1998, 2005; Tomadakis, 1960–1961) and that the foreigners who came to Crete did not penetrate into the countryside until much later and, even then, on a limited scale (Gasparis, 1998, 2005).

Our genetic analysis provides further evidence to address such historical controversies. Our statistical analyses included PCA, ADMIXTURE, IBD, ALDER, and the ChromoPainter/FineSTRUCTURE pipeline. All results from those statistical tools seem to be (broadly) in agreement. That being said, overinterpretation of statistical analyses is a common pitfall (especially when it comes to the ADMIXTURE and PCA) and further analyses using larger sample sizes, more sophisticated statistical tools, and ancient DNA might shed further light on the historical controversies surrounding Crete.

The genetic impact of the Andalusian and the Near Eastern Arabs seems minimal; similarly, the genetic contribution of the Venetians to Cretans is low Tables S6–S8. This might be explained by the geography of Crete, which has many poorly accessible regions, as well as its agricultural and pastoral economy, which is sustained by a large number of small villages and hamlets spread all over the island. The Arab and Venetian conquerors settled mostly in the coastal urban centers and left the rural population intact.

An unexpected insight in the history of Crete was provided by the IBD analysis. The results showed that the Cretans share high IBD with Western (CEU), Central (German, Polish), Northern (CEU, Scandinavian), and Eastern (Ukranian, Russian) European populations. Indeed, in previous studies, there has been an excess of IBD sharing reported between Eastern Europe and the Greeks, while an admixture event has been inferred using Poland as a representative population of the eastern component in the Greek population (Hellenthal et al., 2014; Ralph & Coop, 2013). Especially the results of Hellenthal et al. (2014) used GLOBETROTTER to infer (under a pulse‐admixture model that has the same assumptions as ALDER) that Greek DNA could be described as the mixture of 37% DNA from a Polish‐like source and 63% from a Cypriot‐like source occurring sometime between 718–1138 C.E.; our analyses here support these findings.

These results might reflect past settlements to Crete from Europe. Indeed, Crete was invaded from the North by the Myceneans and the Dorians in prehistoric or early historic times. These were Greek tribes which, together with the Minoans and other prehistoric inhabitants of the island, shaped the genetic structure of the Cretan population. The origin of the Greeks has been debated (see, for example, Drews, 1988; Hooker, 1999; Renfrew, 1987; Wyatt, 1970) and their Indo‐European homelands have been placed in Anatolia (Renfrew, 1987) or in the steppes of Ukraine and Russia (Gimbutas, 1970). Since Crete, Sicily, and Cyprus have been colonized by Greeks in prehistoric or historic times, we investigated whether Sicily and Cyprus also shared higher IBD with European populations; indeed, they both do (Tables S9–S10). A possible interpretation of the IBD results is that they reveal the ancient Greek ancestry of the populations of the three islands. Another interpretation is that the IBD results in the three islands reflect unrelated historical events: the IBD sharing between Sicily and Northern/Eastern Europe may reflect the century‐long occupation of medieval Sicily by the Normans, even though there is no record of extensive Norman settlement in Sicily (Burry, 1963). The IBD sharing between Cyprus and Northern/Eastern Europeans could be explained if, as a result of the expansion in Asia Minor of Turkish states in 12th–13th centuries C.E., Byzantine populations, which included Scythians and Slavs, migrated to Cyprus.

In the fourth to sixth centuries C.E., the Goths invaded the Balkans, settled in Thrace and Moesia (central and northern Balkans) and in areas of Asia Minor, attacked the islands including Crete and Cyprus, devastated Greece under Alaric, and eventually attained considerable Germanic influence on the affairs of the 5th and 6th century Byzantine Empire (Vassiliev, 1980). There is no record by contemporary or later historians of Gothic settlements in Crete. In the sixth and seventh centuries C.E., the Slavs migrated to the Balkans and settled in several areas including parts of the interior of the Greek mainland, less in the coastal regions and in the islands. There is no record by contemporary or later Byzantine or Latin historians of Slavic settlements in Crete. However, an eighth century Arabic manuscript contains a single sentence stating that “the year 934 of the Seleucid era (623 C.E.) Crete and several islands were raided by the Slavs” (Krinov, 1995). Also, as mentioned in this section, one ambiguous sentence in the text of a contemporary historian suggests that following the recapture of Crete from the Arabs in the 10th century C.E., a resettlement policy of various tribes in the depopulated island was instituted by the Byzantines. If the “tribes” that moved to the depopulated Crete included Hellenized Goths (Gothogreeks; Ostrogorsky, 1969; Turtledove, 1982) and Hellenized Slavs (Charanis, 1961) from Asia Minor, the IBD data could be explained.

The results of the ALDER analysis suggest that settlers from the North reached Crete between the third and the 13th centuries C.E. This time frame is compatible with either the Goths reaching Crete in the fourth to sixth centuries or the Slavs in the seventh century or with Byzantines resettling populations on the island the 10th century C.E. We caution, however, that the pulse model of admixture may not capture the history well and does not exclude the possibility of earlier migrations from the north that would be in better agreement with the historical record. Present‐day Greeks have ancestry from the Eurasian steppe at a lower rate than those from Northern Europe (Haak et al., 2015). Our results suggest that at least some of this ancestry may have been introduced not directly from Bronze Age steppe migrations, with historical migrations during the medieval period, long after the first appearance of the Greek language in the Aegean basin. While ancient DNA from the Aegean has not yet been extracted, it is possible that early populations of the region would resemble the genetically homogeneous but geographically dispersed early Neolithic farmers from Anatolia, Central Europe, and Iberia (Haak et al., 2015; Mathieson et al., 2015). Future studies of ancient DNA may provide new insights on how and when the present‐day population of Crete was formed.

## Supporting information


**FIGURE S1** Map of all populations in Tables S2 and S3 except Ashkenazi
**FIGURE S2** Principal component analysis plots of Cretan subpopulations: the top two to five principal components are shown
**FIGURE S3** (a) ADMIXTURE analysis results for the 17 Cretan subpopulations. (b) Dendrogram resulting from the FineSTRUCTURE/ChromoPainter analysis of the Cretan samples, following a chromatic ordering of the Cretan subpopulations based on latitude
**FIGURE S4** Correlation between geographic coordinates (latitude) and the second principal component
**FIGURE S5** Relationships between Crete and neighboring populations
**FIGURE S6** Bootstrap results for Europe, Caucasus, and the Near East
**FIGURE S7** Bootstrap confidence intervals for regions of Europe
**FIGURE S8** Heat map comparing relationships between Crete and Southern European populations
**FIGURE S9** Bootstrap confidence intervals around mean pairwise identity by descent for populations in Table S4
**FIGURE S10** Bootstrap confidence intervals around mean pairwise identity by descent for populations in Table S5
**FIGURE S11** Principal component analysis plots for the Cretans and several European populations
**FIGURE S12** ADMIXTURE analysis results for the Cretans and European populations
**FIGURE S13** ADMIXTURE analysis results for the Cretans and Slavic populations
**FIGURE S14** A network analysis and visualization of the connections between the populations of Main Figure 6(a) as revealed by the top three (panel a) and top five (panel b) principal components
**FIGURE S15** ADMIXTURE analysis results for the Cretans and the Sicilian populations
**FIGURE S16** (a) Dendrogram resulting from the FineSTRUCTURE and ChromoPainter analysis of the Southern European populations, following a chromatic ordering of the populations. (b) Pairwise coincidence matrix generated by the FineSTRUCTURE and ChromoPainter analysis using Southern European populations
**FIGURE S17** Principal component analysis plots for the Cretans and Near Eastern Semitic populations
**FIGURE S18** ADMIXTURE analysis results for the Cretans and the Near Eastern Semitic populations
**FIGURE S19** Principal component analysis results for the Cretans and populations of medieval conquerors and settlers in the island
**FIGURE S20** ADMIXTURE analysis results for the Cretans, the Andalusians, the French, and the Basque
**FIGURE S21** ADMIXTURE analysis results for the Cretans and North African populations
**FIGURE S22** ADMIXTURE analysis results for the Cretans and Near Eastern populations
**FIGURE S23** Principal component analysis plots comparing the Cretan populations with the population of Armenia
**FIGURE S24** ADMIXTURE plot of Cretans and Armenians
**FIGURE S25** ADMIXTURE analysis results for the Cretans, Venetians, and two Italian populations
**TABLE S1** The 17 Cretan subpopulations included in the study
**TABLE S2** Populations used in this study
**TABLE S3** Populations sharing identity by descent with west Crete
**TABLE S4** Populations sharing identity by descent with East Crete
**TABLE S5** Linkage disequilibrium–based inference of admixture times and proportions for the Cretan population using ALDER
**TABLE S6** Amount of Cretan ancestry captured by the Andalusians, the Basques and the French
**TABLE S7** Amount of Cretan ancestry captured by Near Eastern populations
**TABLE S8** Amount of Cretan ancestry captured by the population of Veneto
**TABLE S9** Populations sharing identity by descent with Sicily
**TABLE S10** IBD shared with Cyprus (*n* = 30); populations are sorted alphabetically
