# Supplementary material for: Genetic history of the population of Crete
Source: Ann Hum Genet. 2019 Jun 13;83(6):373–88. doi: 10.1111/ahg.12328 (PMC6851683; doi:10.1111/ahg.12328)
Supplement: Supplementary file 1 — FIGURE S1 Map of all populations in Tables S2 and S3 except Ashkenazi FIGURE S2 Principal component analysis plots of Cretan subpopulations: the top two to five principal components are shown FIGURE S3 (a) ADMIXTURE analysis results for the 17 Cretan subpopulations. (b) Dendrogram resulting from the FineSTRUCTURE/ChromoPainter analysis of the Cretan samples, following a chromatic ordering of the Cretan subpopulations based on latitude FIGURE S4 Correlation between geographic coordinates (latitude) and the second principal component FIGURE S5 Relationships between Crete and neighboring populations FIGURE S6 Bootstrap results for Europe, Caucasus, and the Near East FIGURE S7 Bootstrap confidence intervals for regions of Europe FIGURE S8 Heat map comparing relationships between Crete and Southern European populations FIGURE S9 Bootstrap confidence intervals around mean pairwise identity by descent for populations in Table S4 FIGURE S10 Bootstrap confidence intervals around mean pairwise identity by descent for populations in Table S5 FIGURE S11 Principal component analysis plots for the Cretans and several European populations FIGURE S12 ADMIXTURE analysis results for the Cretans and European populations FIGURE S13 ADMIXTURE analysis results for the Cretans and Slavic populations FIGURE S14 A network analysis and visualization of the connections between the populations of Main Figure 6(a) as revealed by the top three (panel a) and top five (panel b) principal components FIGURE S15 ADMIXTURE analysis results for the Cretans and the Sicilian populations FIGURE S16 (a) Dendrogram resulting from the FineSTRUCTURE and ChromoPainter analysis of the Southern European populations, following a chromatic ordering of the populations. (b) Pairwise coincidence matrix generated by the FineSTRUCTURE and ChromoPainter analysis using Southern European populations FIGURE S17 Principal component analysis plots for the Cretans and Near Eastern Semitic populations FIGURE S18 ADMIXTURE anal [file AHG-83-373-s001.docx]

**SUPPLEMENTARY MATERIALS**

Supplementary Note

Supplementary Figures 1-25.

Supplementary Tables 1-10.

Supplementary References

**Supplementary Note**

**The ethno-geography of Crete (details)**

Crete is a mountainous island and a large number of villages are located in areas which were relatively inaccessible in the past. The eastern-most district, Sitia, is a mountainous and hilly area which in the past was accessible mainly from the south. West of Sitia is a plain and a partly hilly region bordered by Mount Dicte; the district of Ayios Nikilaos occupies the northern and Ierapetra the southern part of this region. Lassithi Plateau is a geographically isolated region in the highlands of Mount Dicte. The northern slopes of Mount Dicte and the coastal plain in the north of it are occupied by the district Neapoli. West of Mount Dicte is Mount Ida and between the two mountains there is a region which extends from the northern to the southern coast of Crete. This region contains the Messara plain, the main agricultural area of the island; the major archaeological sites of Knossos and Phaestos are located in that region. The districts of Viannos and Kasteli are located mostly in the western slopes and in the foothills of mount Dicte while the villages of districts of Harakas, Ayia Varvara and Moires are located mostly in the Messara plain. The districts of Anoya and Perama consist of several villages in the highlands and in the northern slopes of Mount Ida. Spili is located in the South-West slopes of Mount Ida. West of Mount Ida is a mostly mountainous area bordered by the White Mountains. The district of Vamos occupies the north of this area while its center and south as well as the south of White Mountains are occupied by the district of Sfakia. The western-most area of Crete is occupied in the south by the district of Kandanos and in the north by the district of Kissamos. The villages of these districts are located in the western slopes of the White Mountains and, those closer to the coast, in valleys several of which were accessible with difficulty before modern roads were built.

**Supplemental Methods: Meta-analysis of ADMIXTURE output.**

We also used the method developed in (11) in order to meta-analyze the output of ADMIXTURE. Specifically, the proposed method determines, given a target population X and reference populations Y, Z, etc., the amount of overlap between population X and populations Y, Z, etc., as reported by ADMIXTURE.

**Supplemental Results: quantitative assessment of the genetic effects of medieval events.**

To quantify the findings of the various ADMIXTURE analyses, we devised a simple method for meta-analysis of the ADMIXTURE output. As described in the previous section, our approach treats the output of ADMIXTURE as a set of vectors in a K-dimensional space (for a particular value of K between two and eight). Each population is then summarized by a single vector (using PCA) and vector space calculus is used in order to identify the percentage of ancestry of a target population that is captured by one or more reference populations. It is worth noting that our choice to summarize each population by a single vector is akin to computing the mean ADMIXTURE output for a particular population. In most cases, ADMIXTURE returns a homogenous structure for a particular population and thus the top principal component is a good summary of the sample vectors returned by ADMIXTURE.

First, we focused on the ADMIXTURE plot of **Figure S19**, which includes the Cretans, the French, the Andalusians, and the Basque. **Table S6** shows the percentage of the Cretan ancestry (the Cretans are always our target population, denoted by X in the Methods section) that is captured by various combinations of reference populations for values of K between four and eight. In **Table S6** we report results for all values of K as well as the median value in the last column. We first tested the amount of Cretan ancestry that is captured by the Basques and, as expected, the resulting percentage is quite small (median value of 0.2%). We also tested the amount of Cretan ancestry that is captured by the French (median value 1.4%) and the Andalusians, who had the highest median value of 7%. Next, we explored the amount of Cretan ancestry that is captured by the Andalusians after removing the “European” influence (as captured by the French); the resulting median value was 3.1%. The above results indicate that the genetic signature of the Andalusians on the Cretans is very limited, especially if one accounts for the common European genetic signature of both populations. It is worth noting that the Andalusian ancestry in Kissamos, Kandanos, Sfakia, and Anoyia is extremely limited and it appears that these populations had an even smaller number of inter-marriages than the other Cretan subpopulations that we analyzed.

Our next meta-analysis of ADMIXTURE data tested the amount of near-Eastern ancestry for the Cretan samples. Towards that end, we analyzed data from the ADMIXTURE plot in **Figure** **S21** (including the Cretans, the Kurds, the Syrians, the Lebanese, and the Jordanians). As shown in **Table S7**, the amount of Cretan ancestry that is captured by the Near Eastern populations according to our meta-analysis of the ADMIXTURE data is quite limited (median values of 1.2% for the Jordanians, 2.9% for the Lebanese, and 6.5% for the Syrians). The only exception is the Kurdish population, for small values of K (K=4 and K=5). Larger values of K result in considerably smaller values of shared ancestry between the Kurds and the Cretans, with a median of 4.5%.

We also applied our meta-analysis algorithm on the ADMIXTURE plot in **Figure S24** which includes Crete, Veneto, Tuscany, and Italy. As shown in **Table S8**, the amount of Cretan ancestry that is captured by the population of Veneto is quite limited, with a median value of 5.2% (again notice that for K=4 the separation between the Veneto population and the Cretans is not particularly pronounced, at least according to ADMIXTURE; the separation increases significantly at larger values of K). After subtracting the Italian ancestry from the Veneto population, the Cretan ancestry that is captured by the residual vector representing the Veneto population minus the Italian ancestry is practically zero.

**SUPPLEMENTARY FIGURE 1**


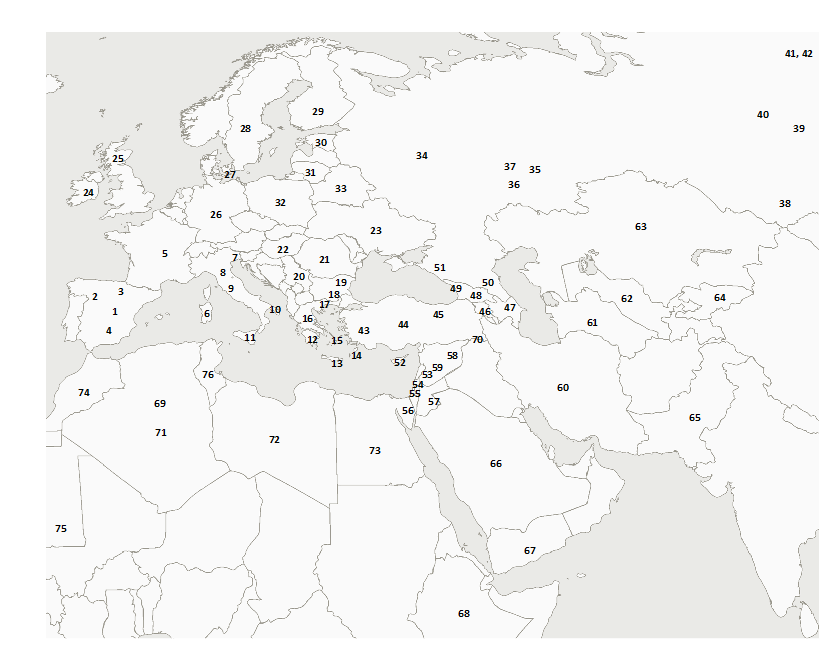


**Supplementary Figure 1.** Map of all populations in Supplementary Tables 2 and 3 except Ashkenazi. Marked location of Russia (34) may not represent the exact sampling locations of the three Russian samples (Russian, Russia_A, and Russians from Table 3). The populations in the figure are (1) Spaniards, (2) IBS, (3) Basque, (4) Andalusians, (5) French, (6) Sardinian, (7) Veneto, (8) Tuscan (including TSI), (9) Italian, (10) Puglia, (11) Sicily, (12) Peloponnese, (13) Crete, (14) Dodecanese, (15) Cyclades, (16) Greece (Sarakatsanoi, Vlachoi Krania, Vlachoi Metsovo), (17) Macedonia, (18) East Rumelia, (19) Bulgarians, (20) Serbia, (21) Romanians, (22) Hungarians, (23) Ukranians, (24) Irish, (25) Orcadian, (26) German, (27) Danes, (28) Swedish, (29) Finns, (30) Estonians, (31) Lithuanians, (32) Polish, (33) Belarusian, (34) Russia, (35) Maris, (36) Mordovians, (37) Chuvash, (38) Altaiains, (39) Selkups, (40) Dolgans, (41) Yakut, (42) Evens, (43) Minor Asia, (44) Cappadocia, (45) Pontos, (46) Armenians, (47) Kumyks, (48) Georgians, (49) Abhkasians, (50) Chechens, (51) Adygei, (52) Cyprus, (53) Lebanese, (54) Samaritans, (55) Palestinian, (56) Bedouin, (57) Jordanians, (58) Syrians, (59) Druze, (60) Iranians, (61) Turkmens, (62) Uzbeks, (63) Kazakhs, (64) Kyrgyzians, (65) Kalash, (66) Saudis, (67) Yemenese, (68) Ethiopians, (69) Mozabite, (70) Kurds, (71) Algeria, (72) Libya, (73) Egypt, (74)
Morocco, (75) Western Sahara (Sahara Occidental), (76) Tunisia.

**SUPPLEMENTARY FIGURE 2**

| 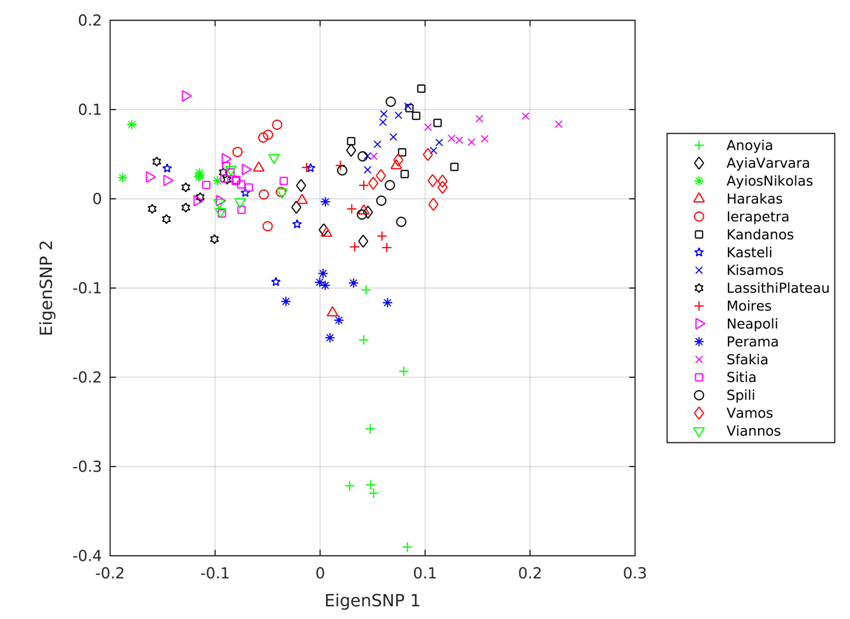  **a.** |
| --- |
| 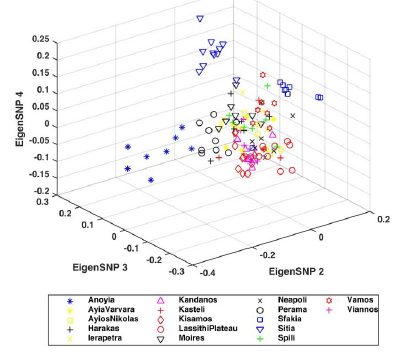  **b.**  **c.** |
| 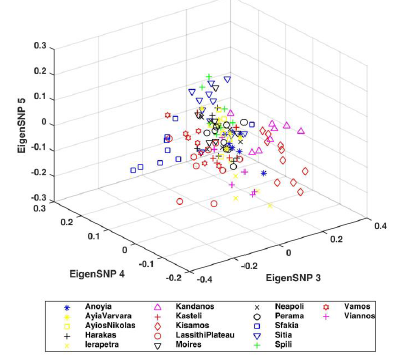 |

**Supplementary Figure 2.** PCA plots of Cretan subpopulations: the top two to five principal components are shown.

1. Results of PCA analysis (two-dimensional) of the 17 Cretan subpopulations.
2. Sfakia show a pronounced separation when the fourth principal component is included. Additionally, Perama is now separated.
3. Little additional structure seems to be revealed by the fifth principal component.

| **SUPPLEMENTARY FIGURE 3A** |
| --- |


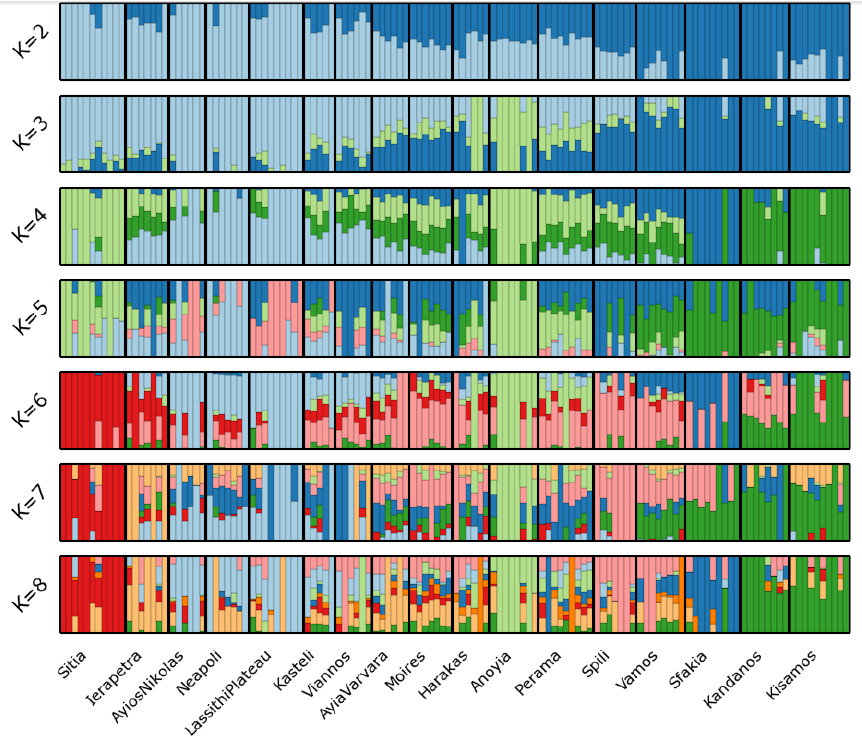


**Supplementary Figure 3A.** ADMIXTURE analysis results for the 17 Cretan subpopulations. A range from two to eight hypothetical ancestral populations (K) is used in the analysis. The subpopulations have been ordered according to geographical location. The far left represents the population at the eastern side of Crete (Sitia) and the far right represents the population at the western side of Crete (Kissamos). Notice that for values of K equal to two and three, a cline appears in the genetic distribution that faithfully reflects the geography of Crete. Also notice the characteristic structures of several sub-populations: Sitia (K=4…8); Anoyia (K=3…8); Lassithi (K=5…8); Sfakia (K=8); the two most western populations, Kissamos and Kandanos (K=8).

**SUPPLEMENTARY FIGURE 3B**

**(see next page)**

**Supplementary Figure 3B.** Dendrogram resulting from the FineSTRUCTURE/ChromoPainter analysis of the Cretan samples, following a chromatic ordering of the Cretan subpopulations based on latitude. We observe that populations are clustered mostly based on relative latitude. We do not observe co-clustering of populations at the "endpoints" of the Crete, although some samples from central Crete are clustered with some of the populations at the "endpoints" of Crete. The isolated population of Sfakia seems to be closely related to the population in the Vamos area; it is also weakly clustered with some Sitia and Kasteli samples. Samples from Central Crete lie in between population at the two latitudinal ends of Crete. A high-resolution version of Supplementary Figure 3b is available at http://www.drineas.org/Crete/S3b.png.


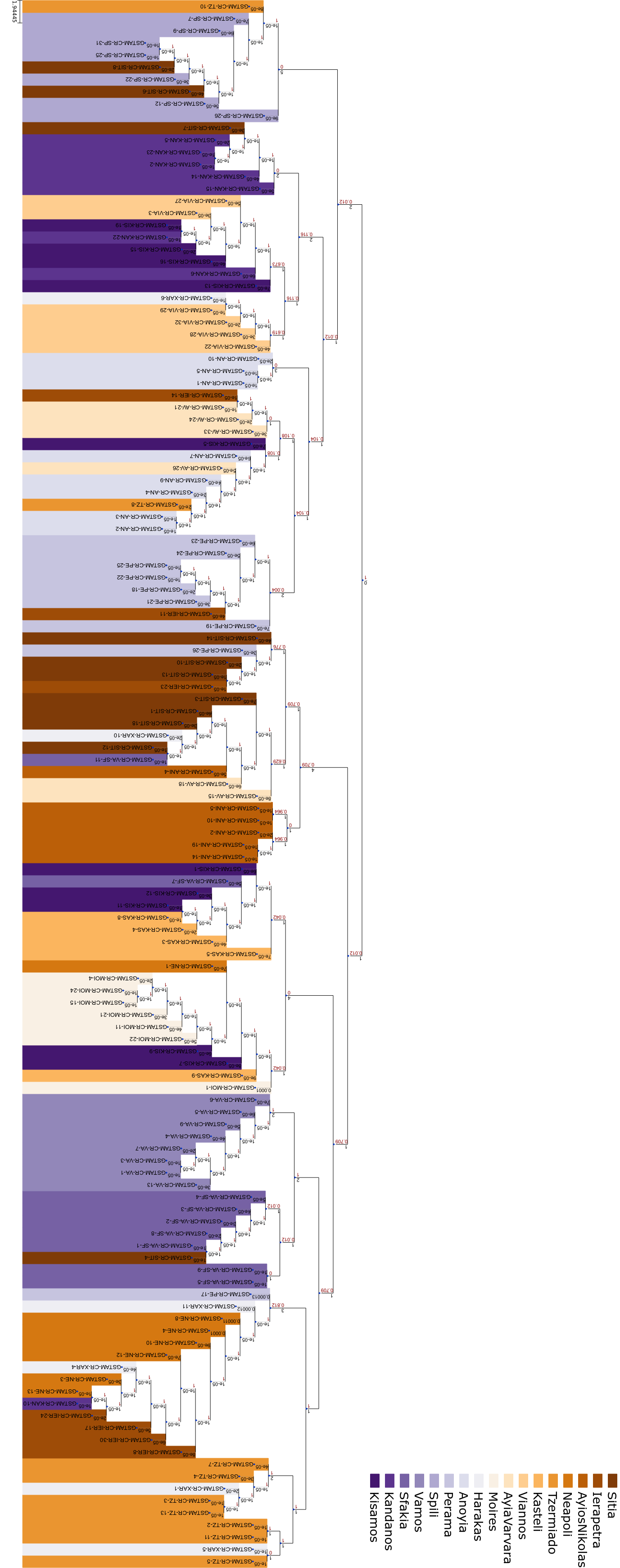


**SUPPLEMENTARY FIGURE 4**


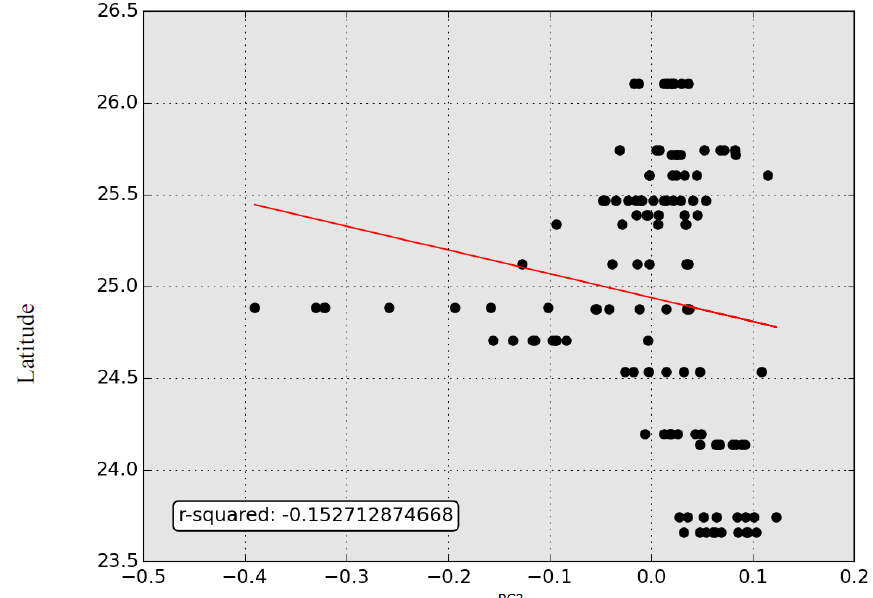


**Supplementary Figure 4.** Correlation between geographic coordinates (latitude) and the second principal component. Notice that in contrast to the east to the west axis (Figure 2B), the north to south axis is not captured well by PCA. This is not surprising given the geography of the Cretan island and how narrow the North to South axis of the island is.

**SUPPLEMENTARY FIGURE 5**

| 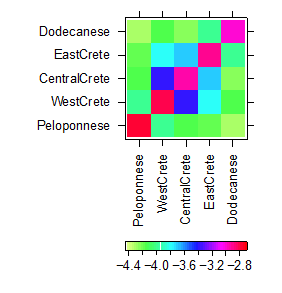  **a.** |  | 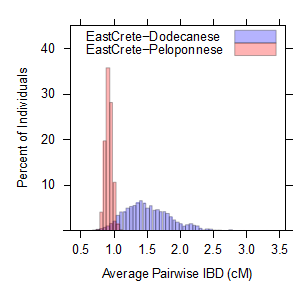  **b.** |
| --- | --- | --- |
| 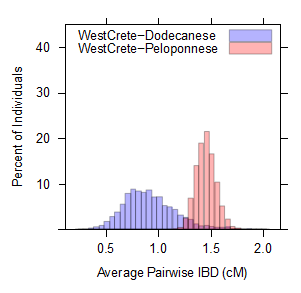  **c.** |  | 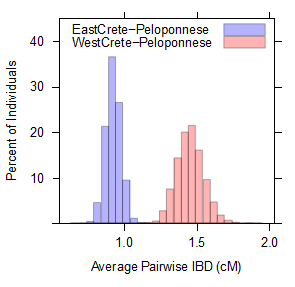  **d.** |

**Supplementary Figure 5.** Relationships between Crete and neighboring populations.

| **a.** | Relationships between Eastern, Central and Western Crete with Dodecanese and Southern Peloponnese. Heat map of the base 10 logarithm of the average proportion of genome shared IBD between a pair of individuals in the specified populations. Higher values (less negative; towards red end of color scale) indicate higher IBD sharing. |
| --- | --- |
| **b.-d.** | Histogram of bootstrapped values of average pairwise IBD sharing (cM) between the specified populations. |

**SUPPLEMENTARY FIGURE 6**


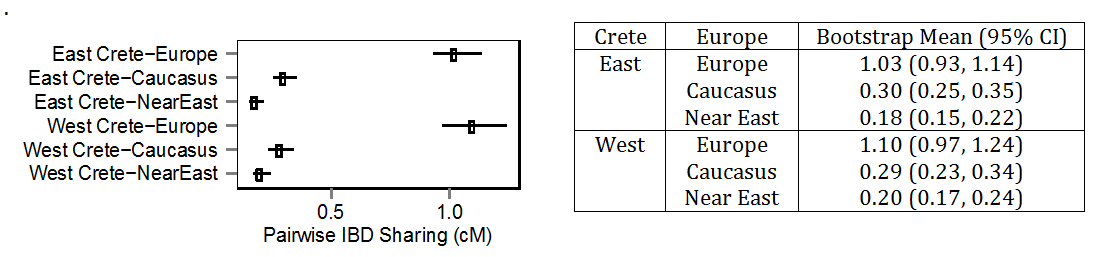


**Supplementary Figure 6.** Bootstrap results for Europe, Caucasus, and the Near East. Europe includes Belarusians, Estonians, Germany, Lithuania, Orcadians, Poland, and Sweden. Caucasus includes Georgia, Armenia, Abhkasians, and Adygei. Near East includes Bedouin, Druze, Jordanians, Palestinians, Samaritans, and Syrians. The circle in the plot indicates the mean across bootstrap samples of the average pairwise IBD between the populations. The line indicates the 95% confidence interval. Based on average pairwise IBD, both East and West Crete are significantly more closely related to Europe than to Caucasus or the Near East.

**SUPPLEMENTARY FIGURE 7**


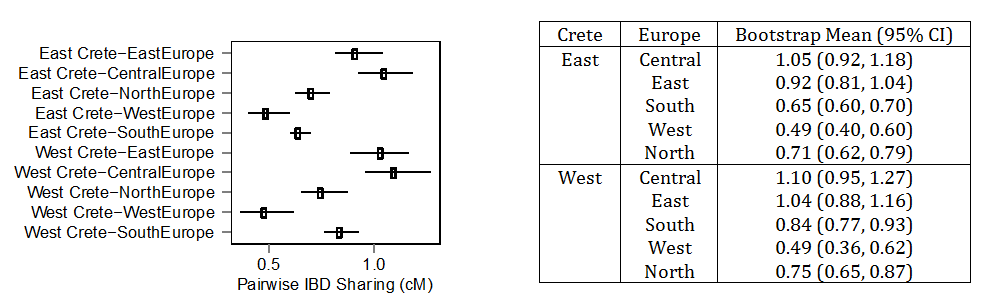


**Supplementary Figure 7.** Bootstrap confidence intervals for regions of Europe. East Europe includes Chuvash and Russia. Central Europe includes Hungary and Ukraine. South Europe includes Sicily, Peloponnese, Serbia, Veneto, Tuscany, Andalusia, Basque, Iberia, Italy, Macedonia, and Sardinia. North Europe includes Denmark, Finland, and Ireland. West Europe includes just France. The circle in the plot indicates the mean across bootstrap samples of the average pairwise IBD between the populations. The line indicates the 95% confidence interval. Based on average pairwise IBD, both East and West Crete are most related to eastern and central Europe, then northern and southern Europe, and both are least related to western Europe (France).

**SUPPLEMENTARY FIGURE 8**


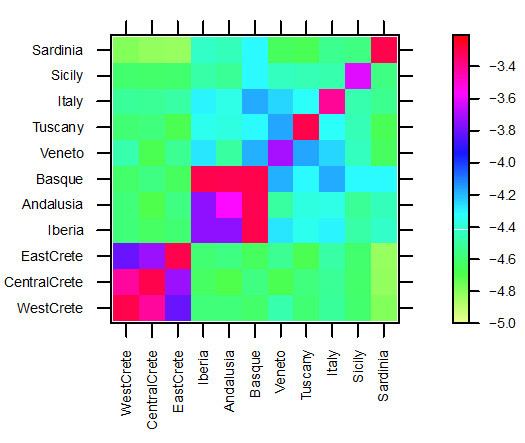


**Supplementary Figure 8.** Heat map comparing relationships between Crete and Southern European populations. Plotted values are the base 10 logarithm of the average proportion of genome shared IBD between a pair of individuals in the specified populations. Higher values (less negative; towards red end of color scale) indicate higher IBD sharing.

**SUPPLEMENTARY FIGURE 9**


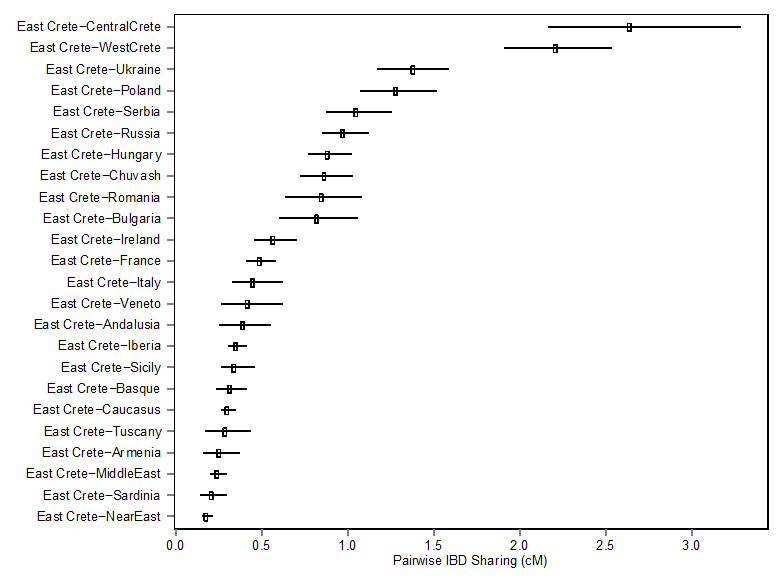


**Supplementary Figure 9.** Bootstrap confidence intervals around mean pairwise IBD for populations in Table S4. The circle in the plot indicates the mean across bootstrap samples of the average pairwise IBD between the populations. The line indicates the 95% confidence interval.

**SUPPLEMENTARY FIGURE 10**


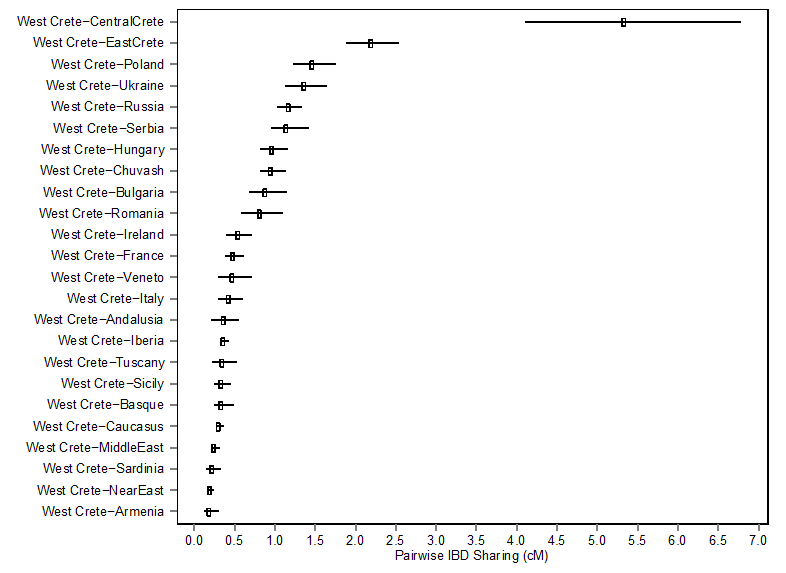


**Supplementary Figure 10.** Bootstrap confidence intervals around mean pairwise IBD for populations in Table S5. The circle in the plot indicates the mean across bootstrap samples of the average pairwise IBD between the populations. The line indicates the 95% confidence interval.

**SUPPLEMENTARY FIGURE 11**

| 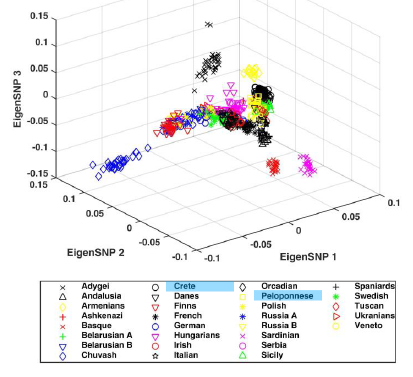  **a.** | 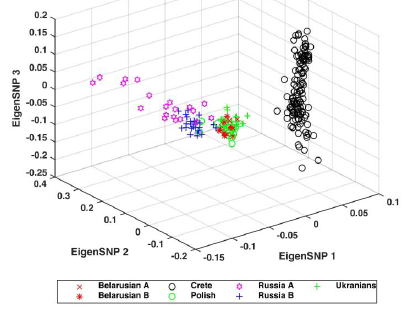  **b.** |
| --- | --- |
| 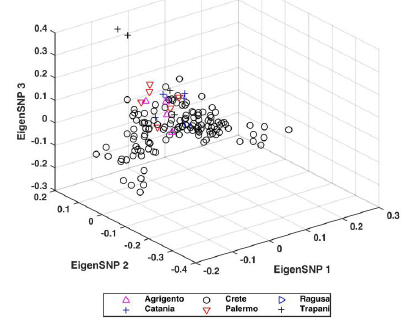  **c.** |  |

**Supplementary Figure 11.** PCA plots for the Cretans and several European populations. The first three principal components are shown.

1. PCA plot of Crete and Europe. Highlighted in blue shade are the labels of the Greek populations of Crete and Peloponnese. There is a north to south cline along the first eigenvector, with the north appearing on the left side and the south on the right side of the plot. The third eigenvector captures the distance of the Armenians and the Adygei, two populations from Caucasus, as well as some of the Hungarians. The Sardinians and the Basque are located outside the European cluster in both the first and third eigenvectors, while the Chuvash are outside the cluster in the second and third eigenvectors. The Cretans overlap with Sicily and also have some overlap with the Ashkenazi and the Peloponnese.
2. PCA plot for the Cretans and the Slavic populations. The first eigenvector captures the genetic distance between the Cretans and the Slavs. The second eigenvector shows the difference between the Slavs, with Russians being the most diverse population across the eigenvector. The third eigenvector captures the diversity between the Cretans. In all three eigenvectors, the Slavic populations are completely distinct from the Cretans.
3. PCA plot for Cretans and Sicily. There is an overlap between most of the Sicilians with the Cretan
    samples. With the exception of two samples from Trapani, the Sicilians seem to be in the same cluster as the Cretans.

**SUPPLEMENTARY FIGURE 12**


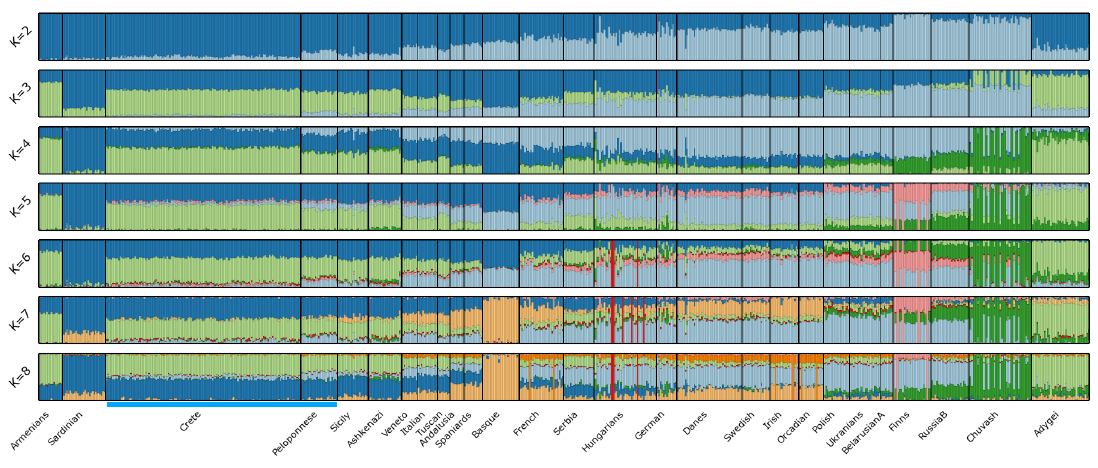


**Supplementary Figure 12.** ADMIXTURE analysis results for the Cretans and European populations. A range of two to eight hypothetical ancestral populations (K) is used. The blue color bar underneath the plot over the labels denotes the two Greek populations in the analysis. The two Greek populations show a great degree of resemblance. There is a south-north cline that can be observed for all values of K, and an east to west cline after K gets larger than three. There is a minimal amount of genetic flow from the Eastern Europeans to the Cretans that becomes even less pronounced for values of K larger than seven. As K becomes larger than seven, the genetic component of the Basques makes the Cretans more distinct than the other Southern European populations.

**SUPPLEMENTARY FIGURE 13**


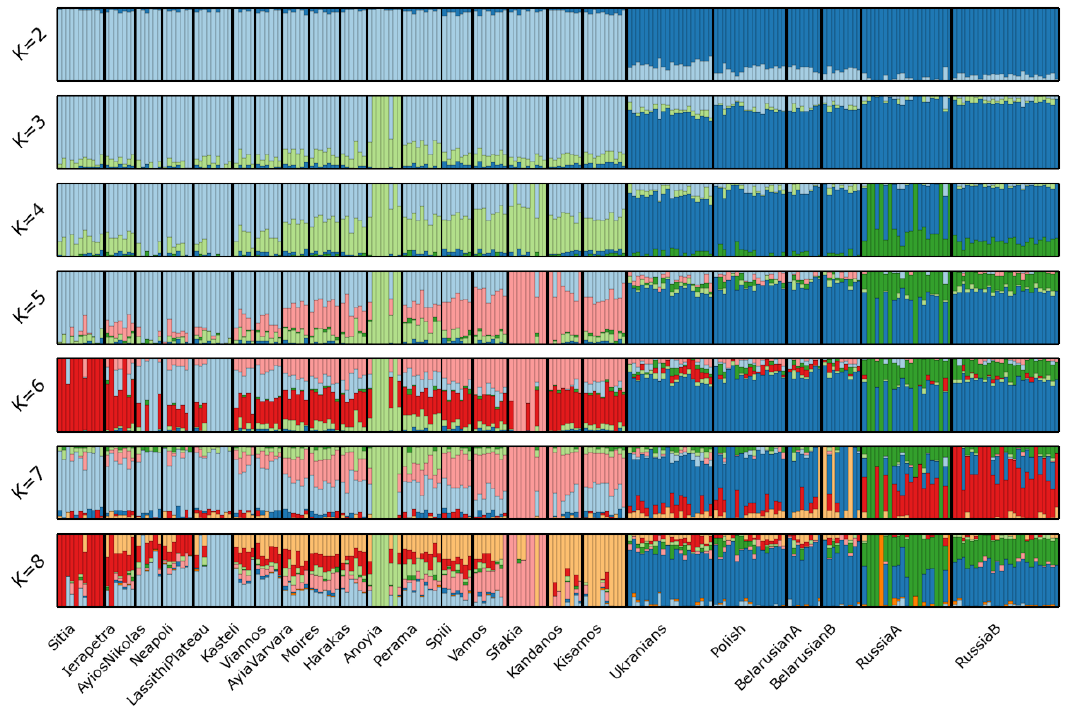


**Supplementary Figure 13.** ADMIXTURE analysis results for the Cretans and Slavic populations. A range of two to eight hypothetical ancestral populations (K) is used. There is a distinct difference between the Slavs and the Cretans that is highlighted for every value of K. As K increases, stratification within the Cretans and the Slavs can be simultaneously detected. The genetic isolation of select Cretan populations gets more pronounced than the internal substructure of the Slavs. We observe substructure in the Russian population for values of K larger than three. There is a low level of gene flow from Slavic populations to all the Cretan subpopulations that can be observed for K between two and four. For values of K between five and eight, the gene flow seems to be lesser in the subpopulations of Sfakia, Lassithi, and Anoyia.

**SUPPLEMENTARY FIGURE 14**

| 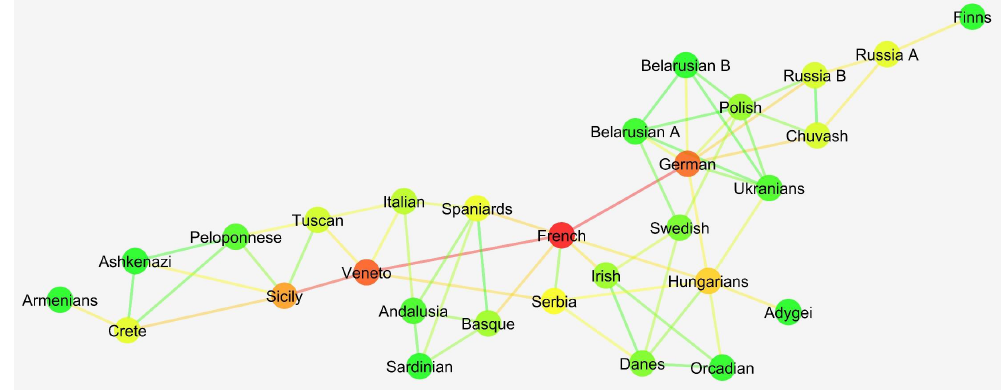 |
| --- |
|  |
| 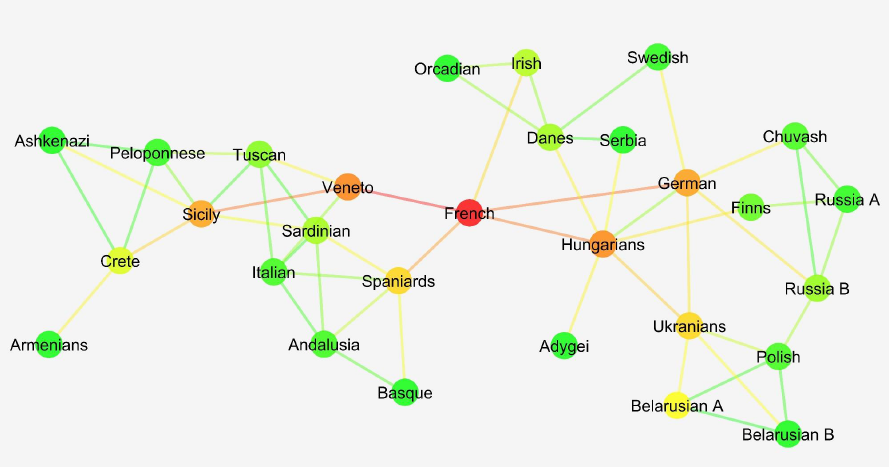 |

**Supplementary Figure 14.** A network analysis and visualization of the connections between the populations of Main Figure 6A as revealed by the top three (panel **A**) and top five (panel **B**) principal components. The network was formed by identifying nearest neighbors of each individual outside its populations of origin. Thicker edges represent stronger genetic relationships between pairs of populations, while warmer colors indicate high centrality of the respective nodes. It is clear that Cretans are connected to Southern European populations and are far away from Northern European populations.

**SUPPLEMENTARY FIGURE 15**


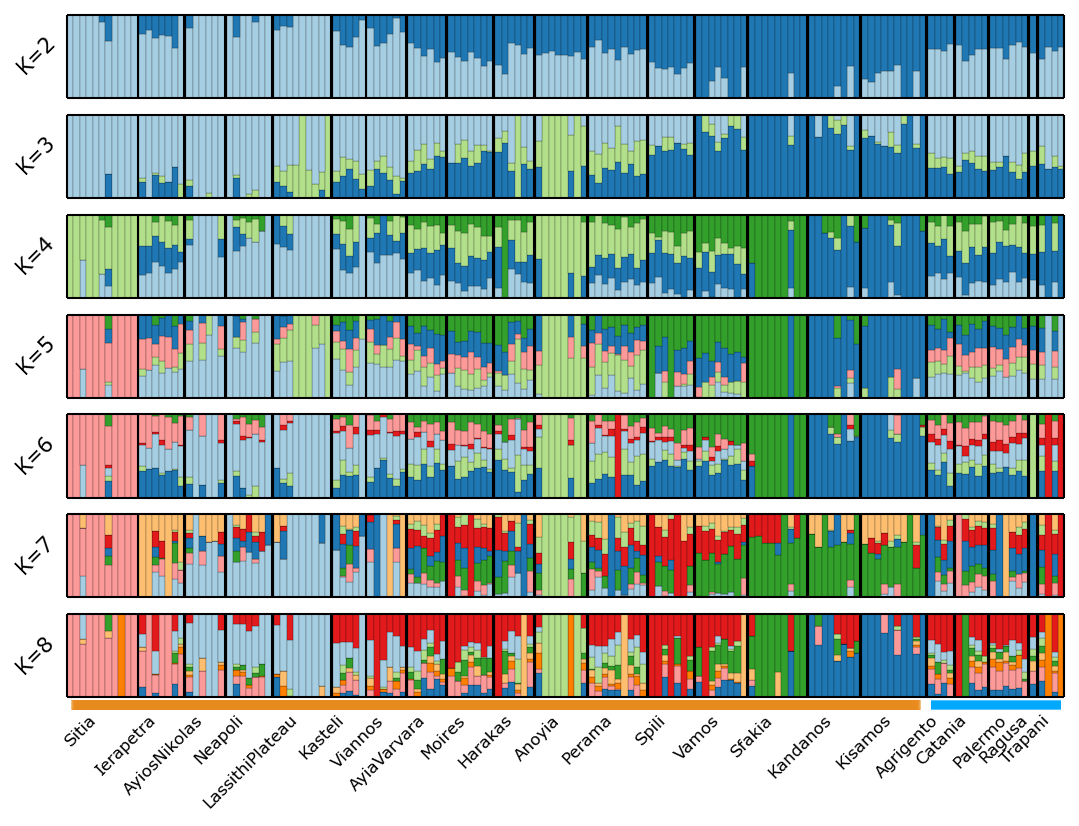


**Supplementary Figure 15.** ADMIXTURE analysis results for the Cretans and the Sicilian populations. The orange color bar denotes Cretan populations, the blue color bar denotes Sicilian populations. A range of two to eight hypothetical ancestral populations (K) is used. There is no clear differentiation between the Cretans and the Sicilians. The more K increases, the more the Sicilians follow the patterns of the Cretans with internal substructure becoming more prominent in each individual.

**SUPPLEMENTARY FIGURE 16**

**(see next two pages)**

**Supplementary Figure 16a.** Dendrogram resulting from the FineSTRUCTURE and ChromoPainter analysis of the Southern European populations, following a chromatic ordering of the populations. We observe Iberian samples forming a large cluster of their own, next to the Sicilian and Venetian clusters. Some Sicilian and Basque samples seem to lie between the Iberian samples. Andalusian samples mostly reside next the Basque samples, into the second major clade, which is closer to the Cretans. Cretan samples are split between the two major clades of the dendrogram, forming a Cretan-dominated subclade. Some Sicilian samples interject the Cretan clade, while the Italian and Tuscan samples form mostly their own clade. Interestingly, Sardinian samples tend to form clades that are somewhat close to the Western Cretan samples. A high-resolution version of Supplementary Figure 16a is available at http://www.drineas.org/Crete/S16a.png.

**Supplementary Figure 16b.** Pairwise coincidence matrix generated by the FineSTRUCTURE and ChromoPainter analysis using Southern European populations. Similar to the dendrogram of the previous figure, we can discern that Iberia forms a cluster of its own, showing genetic relationship with some Sicilian and Basque samples, and minimal relationship with Cretan samples. Two subclusters of the Cretan samples can be seen: one involves the Central and Eastern Crete populations and one that involves Western and Central Crete populations. East and Central Crete also appear to share some genetic background with Sicilian samples. The Andalusian samples do not exhibit genetic sharing with the Iberian samples, but seem to share segments with Sardinians, Tuscan, Basque, and Cretan populations. Sardinians share genetic segments mostly with the Kisamos samples. These results are in line with the dendrograms generated by the FineSTRUCTURE and ChromoPainter analysis. A high-resolution version of Supplementary Figure 16b is available at http://www.drineas.org/Crete/S16b.png.


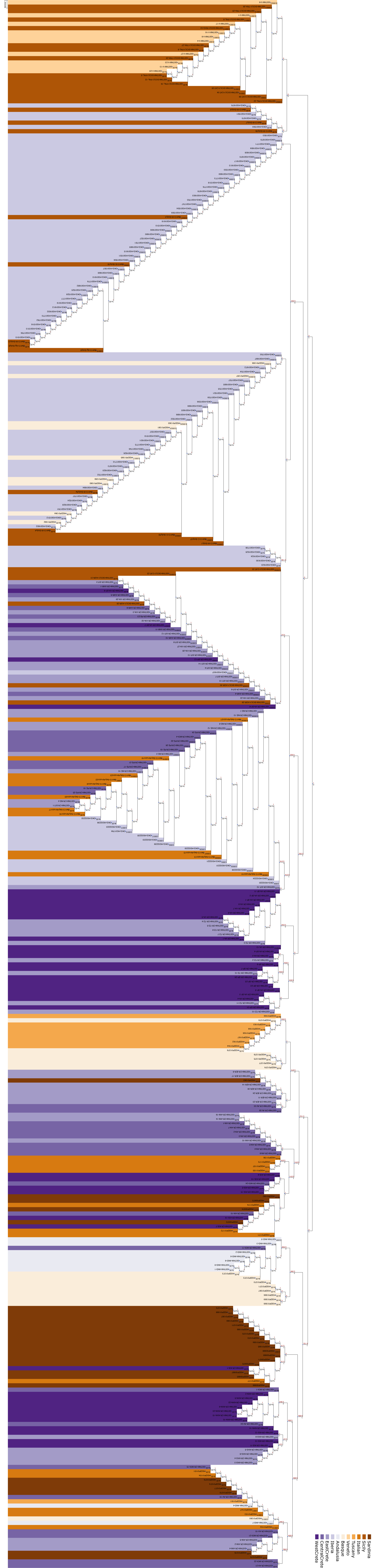
 **S16a**


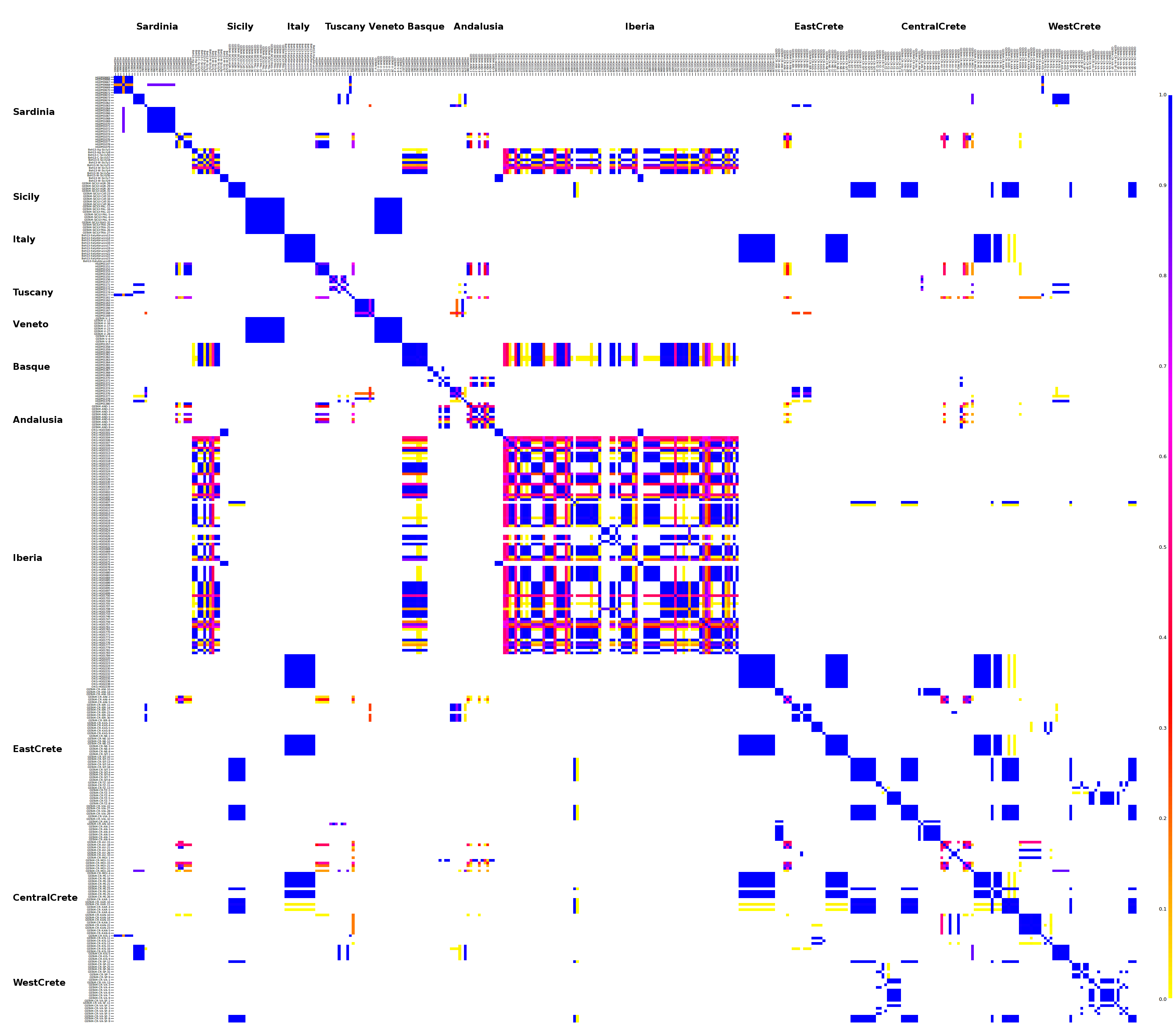


**S16b**

**SUPPLEMENTARY FIGURE 17**


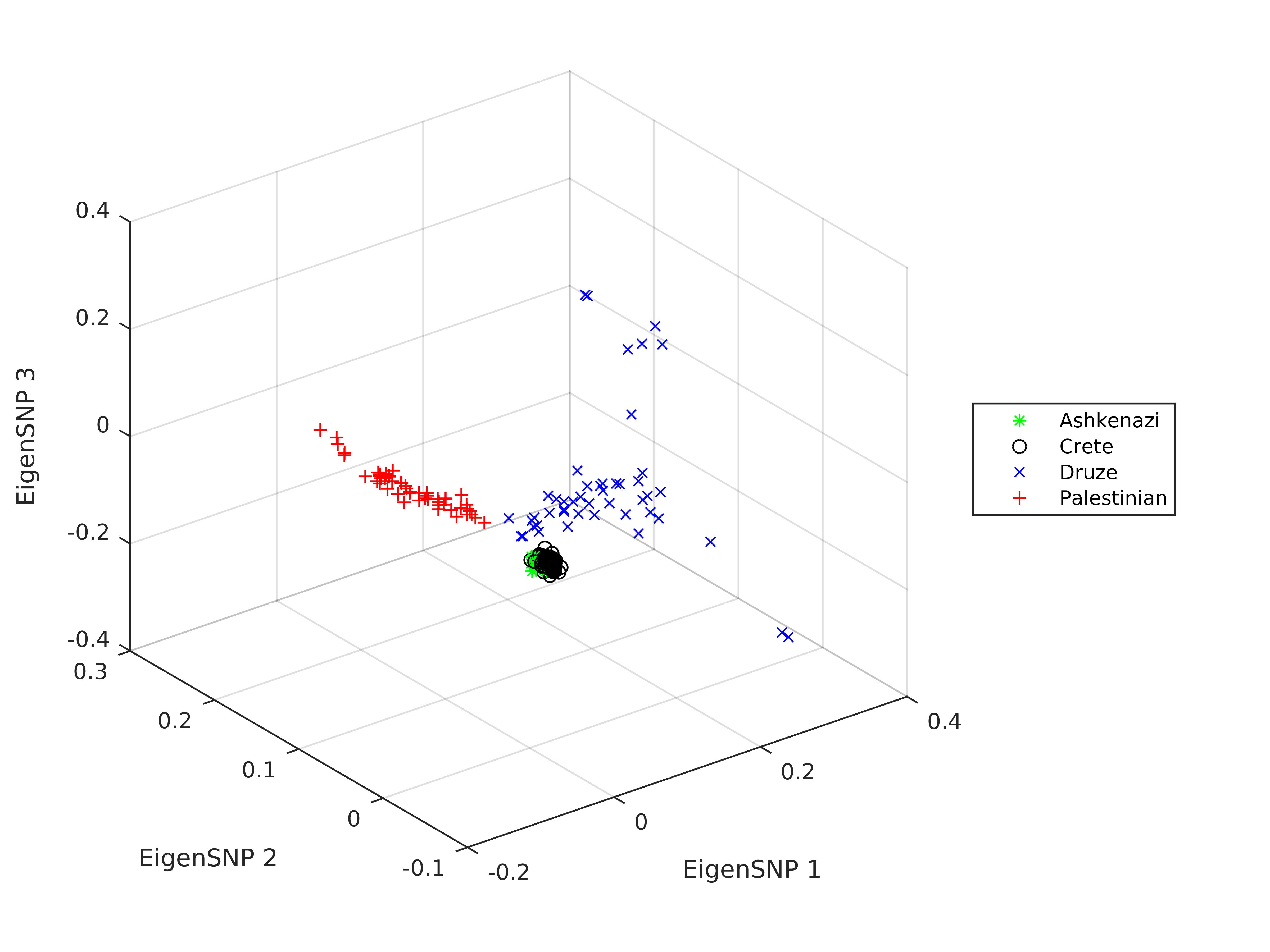

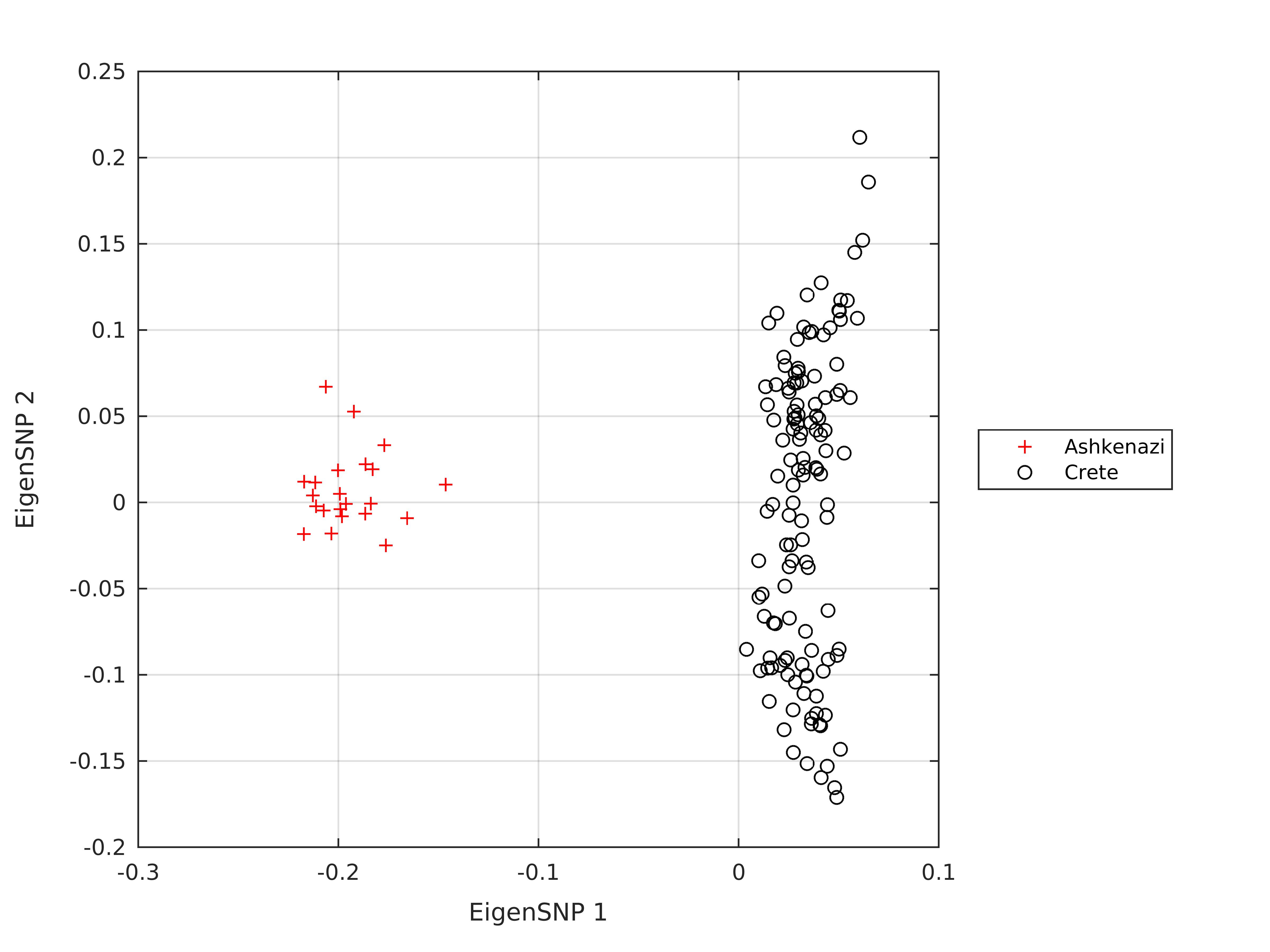


**a.**

**b.**

**d.**

**c.**


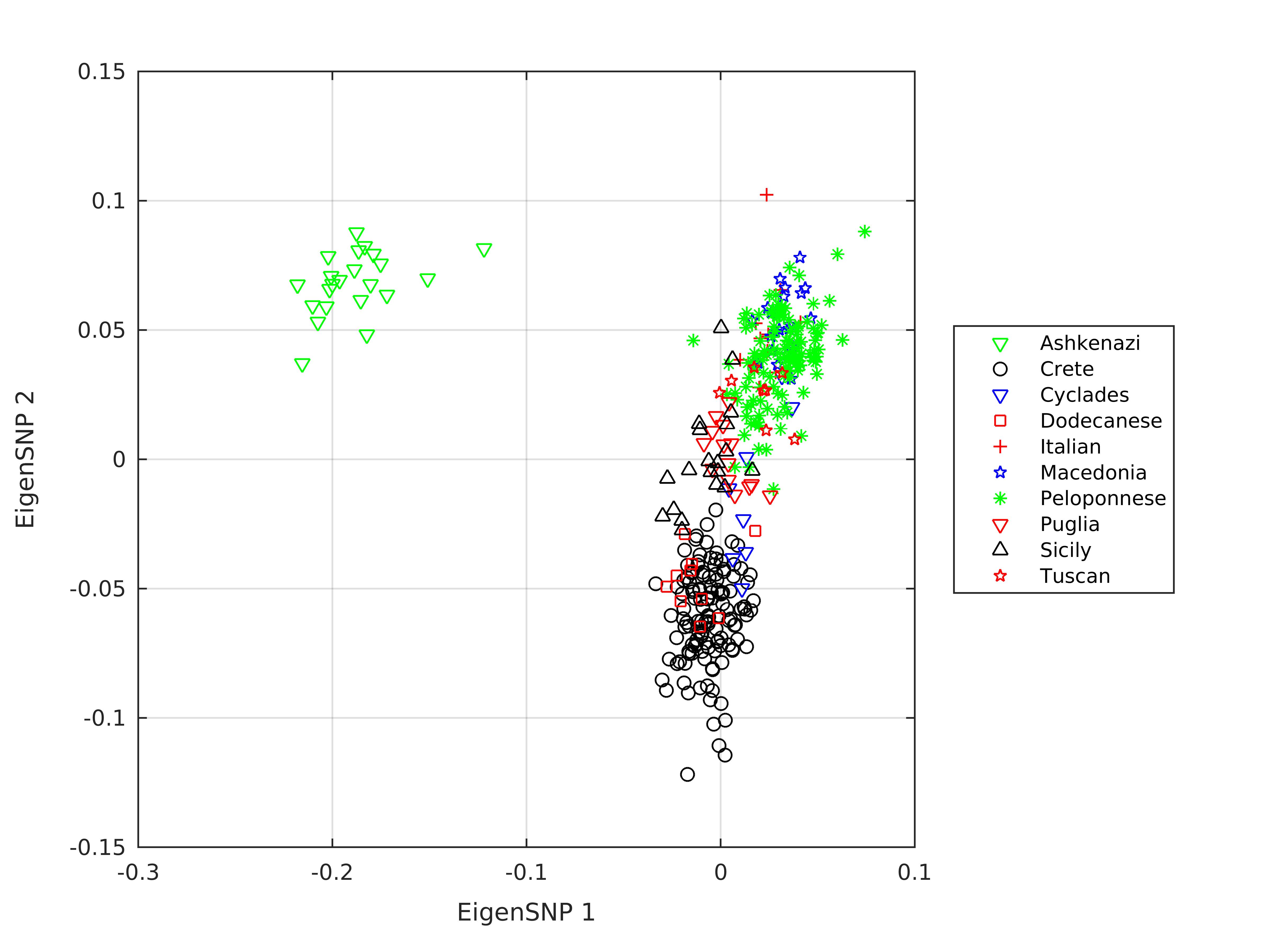

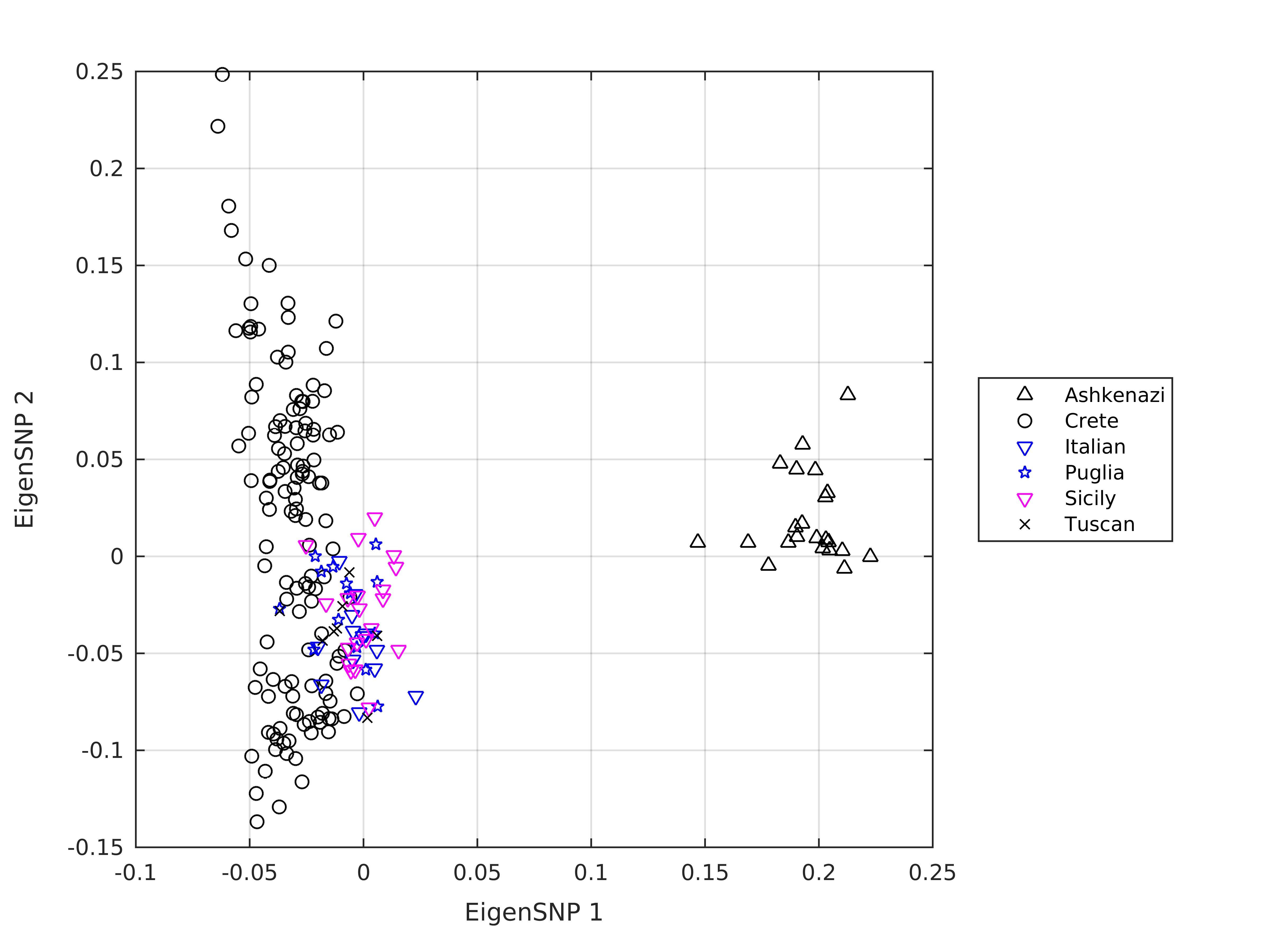


**Supplementary Figure 17.** PCA plots for the Cretans and Near Eastern Semitic populations. The first three principal components are shown.

1. PCA plot of Cretans and the Ashkenazi, Palestinian, and Druze populations. The first two principal components are driven by the variation in the Palestinian and Druze populations. In this analysis, Ashkenazi and Cretans cluster very closely with each other.
2. PCA plot for the Cretans and the Ashkenazi. The Cretans appear to be distinct from the Ashkenazi, with the first component capturing the cross-population differences, while the second component captures the internal variation in both populations, but mainly in Crete.
3. PCA plot for Cretans, other Greeks, Italians and Ashkenazi. The Greeks cluster with the Italians, while forming a gradient that joins the Peloponnese with Cretans. The Ashkenazi continue to be distinct in this analysis.
4. PCA plot for Cretans, Italians and Ashkenazi. The Cretans cluster with the Italians, while the Ashkenazi are distinct from this cluster.

**SUPPLEMENTARY FIGURE 18**


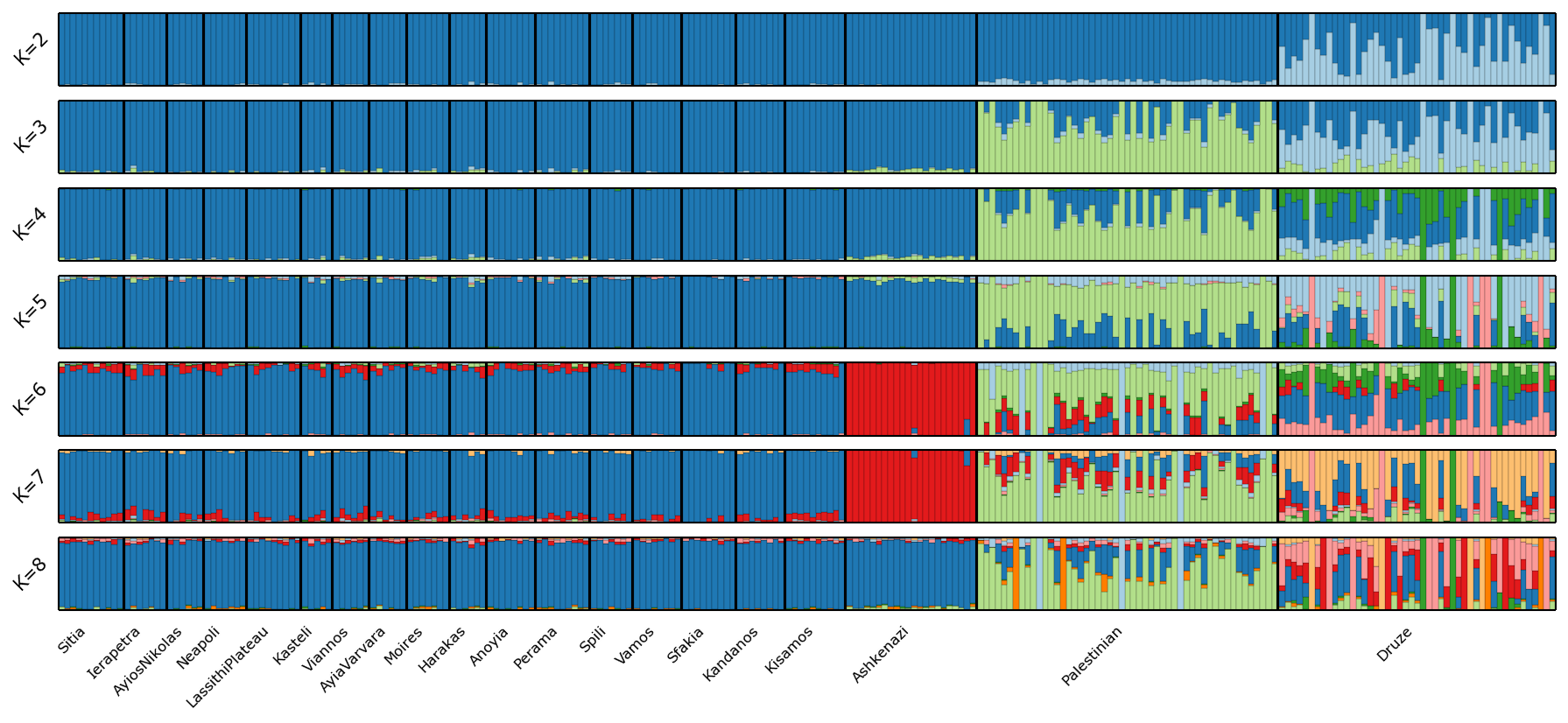


**Supplementary Figure 18.** ADMIXTURE analysis results for the Cretans and the Near Eastern Semitic populations. A range of two to eight hypothetical ancestral populations (K) is used. There is an early differentiation between the Cretans and the other populations. As K increases we can see the substructure mainly of the other populations, while the Cretans remain uniform. There appear to be some signals of gene flow between the Ashkenazi and the other populations. Interestingly, K6 and K7 clearly differentiate the Cretans with the Ashkenazi, while K8 returns to the earlier pattern.

**SUPPLEMENTARY FIGURE 19**

| 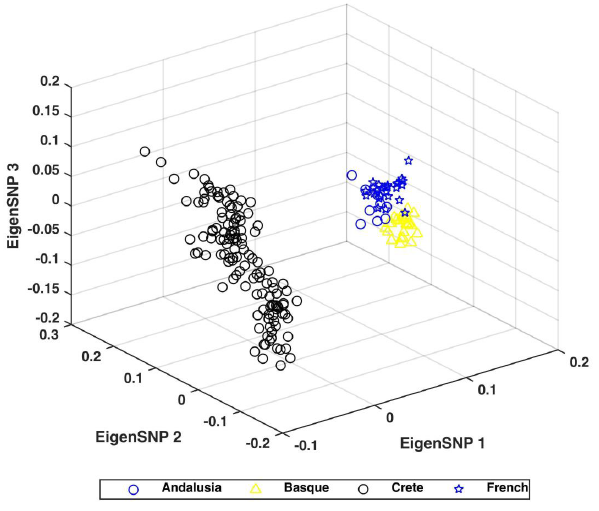  **a.** | 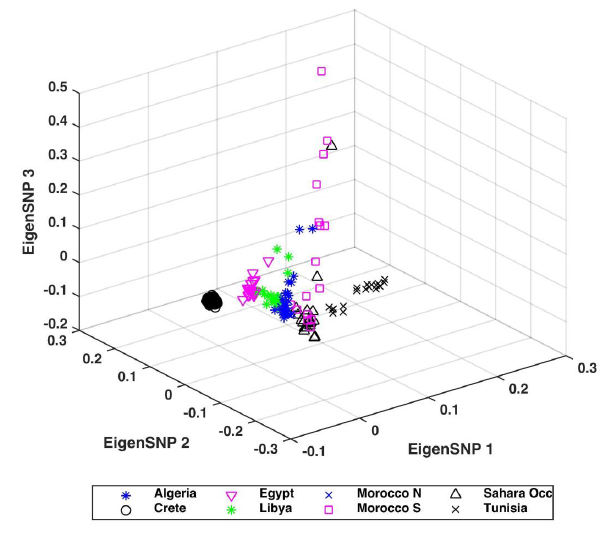  **b.** |
| --- | --- |
| 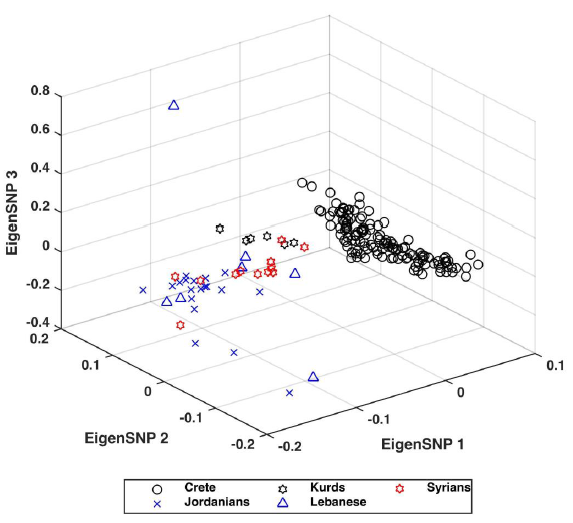  **c.** | 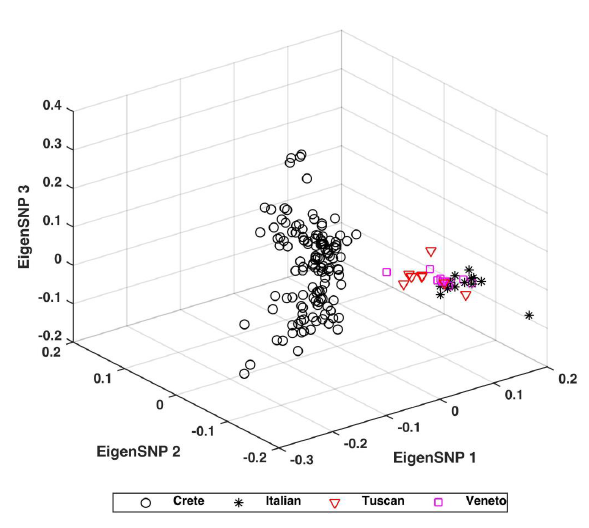  **d.** |

**Supplementary Figure 19.** PCA results for the Cretans and populations of medieval conquerors and settlers in the island. The first three principal components are shown.

1. Comparisons of Cretans with Andalusians. Basque and the French are also included in the graph. The first and third eigenvectors show a clear distinction between the Cretans and Andalusians.
2. PCA plot for the Cretans and the North African populations. The genetic diversity of the North African coast dominates the first two principal components. The first eigenvector captures the geographic and genetic distance from the Cretans. The second eigenvector shows a differentiation of the Moroccan populations, with the Berbers appearing in the lower part of the plot. The third eigenvector captures the diversity of the non-Berber Africans.
3. PCA plot for the Cretans and the Near Eastern populations. The first eigenvector effectively differentiates the Cretans from the Near Eastern populations, while the second eigenvector captures the within-Crete diversity of the populations. The Kurds, along with the Syrians are located close to the Cretans, while the diverse Lebanese and Jordanians are spread over a large area. The third eigenvector captures the diversity of the Lebanese samples.
4. PCA results for the Cretans and the Venetians. Tuscans and Italians sample are also included in the graph. All three eigenvectors capture the genetic diversity and the distance from the Venetians. Some dispersion, reminiscent of the geographical distribution of the plotted population, can be observed for the Italians, with the Tuscan and the Venetians being the closest populations to the Cretan cluster.

**SUPPLEMENTARY FIGURE 20**


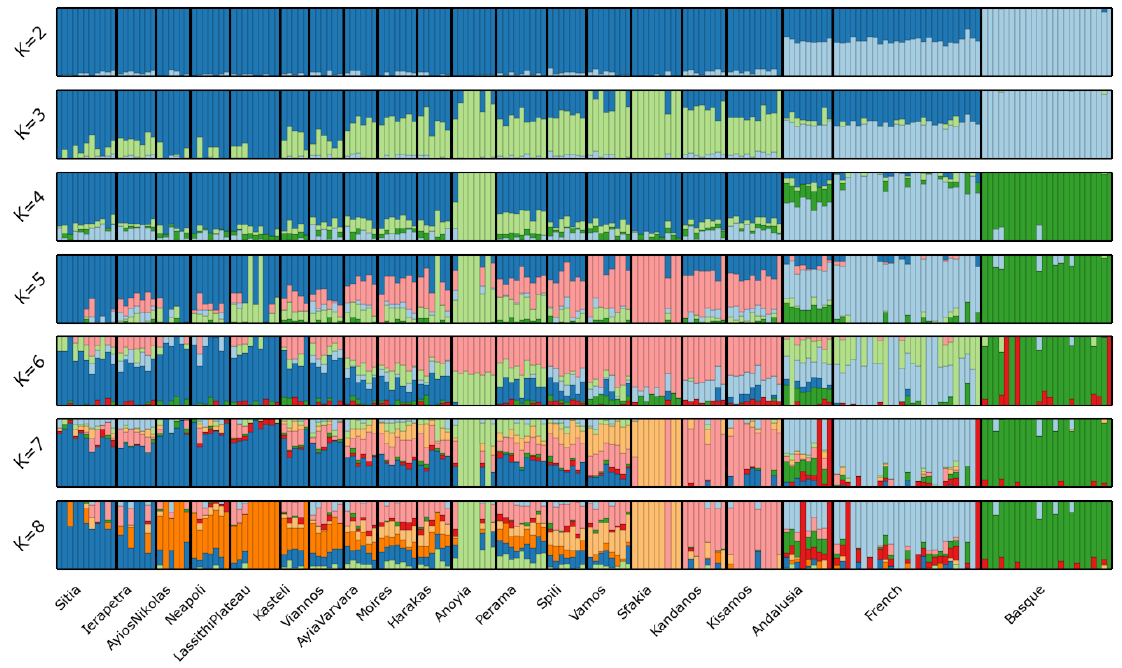


**Supplementary Figure 20.** ADMIXTURE analysis results for the Cretans, the Andalusians, the French, and the Basque. A range of two to eight hypothetical ancestral populations (K) is used. There is an early differentiation between the Cretans and the other populations. As K increases we can see the substructure mainly of the Cretans and the Andalusians, but with some signals of flow between the Cretans and the other populations.

**SUPPLEMENTARY FIGURE 21**


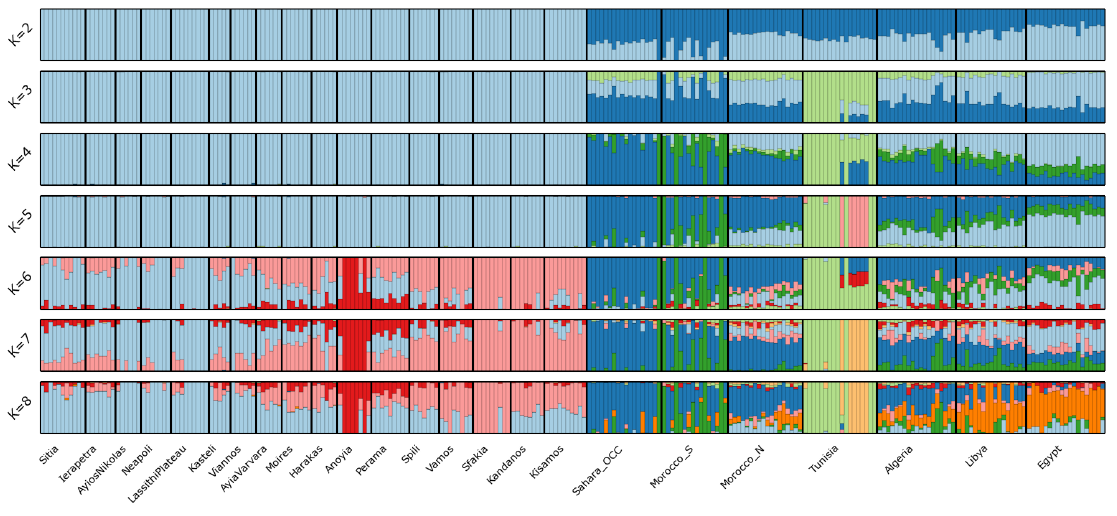


**Supplementary Figure 21.** ADMIXTURE analysis results for the Cretans and North African populations. A range of two to eight hypothetical ancestral populations (K) is used. The North African population substructure dominates the plot. For values of K larger than six, the substructure of the Cretans becomes apparent, and they become completely distinguished from the North Africans.

**SUPPLEMENTARY FIGURE 22**


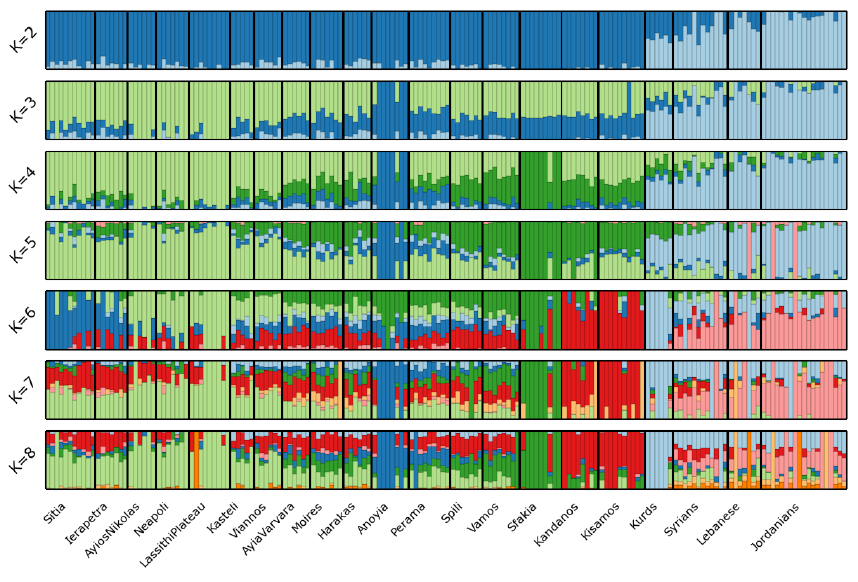


**Supplementary Figure 22.** ADMIXTURE analysis results for the Cretans and Near Eastern populations. A range of two to eight hypothetical ancestral populations (K) is used. The substructure of the Cretans becomes apparent even at low values of K. The substructure of the Near Eastern populations becomes apparent for values of K larger than six. There is a signal of genetic flow from Near East to Crete for all values of K; this signal is minimal at the subpopulations in the west side of Crete, relatively low in the eastern populations, and more pronounced in the central Cretan populations, with the exception of Anoyia. We note that the subpopulation of Sfakia shows the lowest signal of shared ancestry with Near Eastern populations.

**SUPPLEMENTARY FIGURE 23**

**a.**


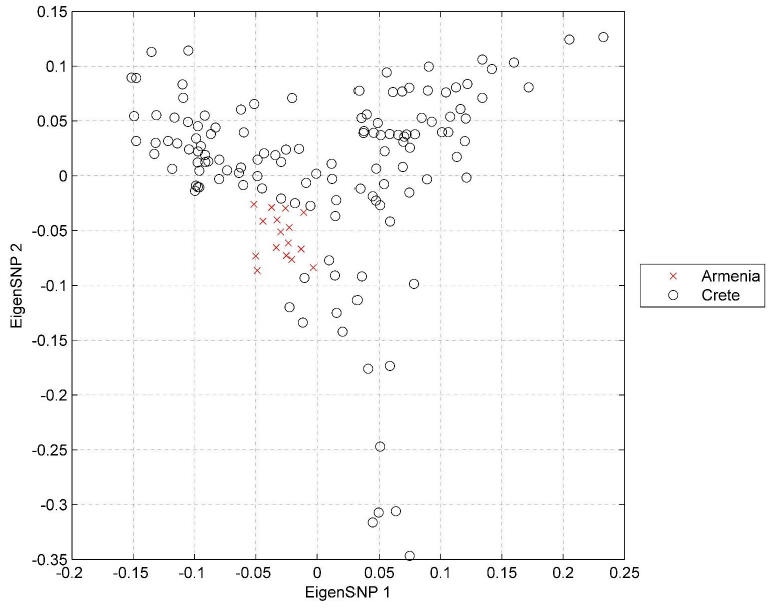


**b.**


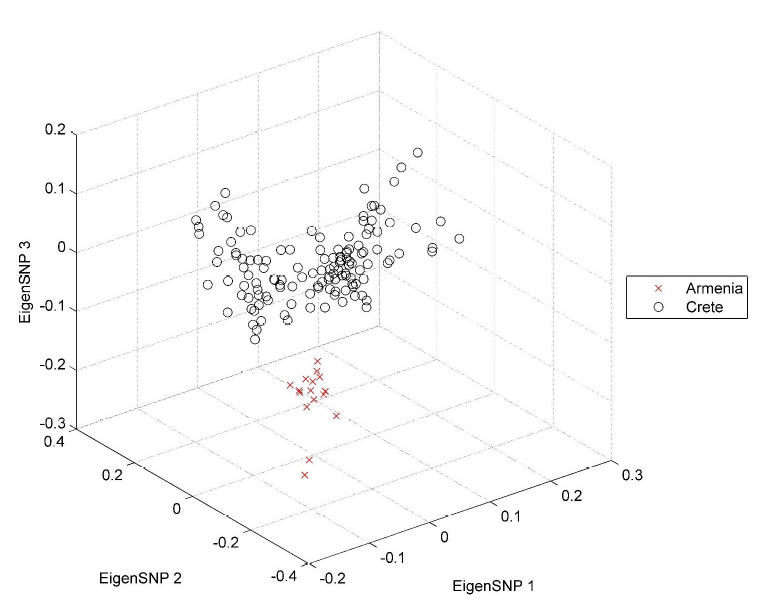


**Supplementary Figure 23.** PCA plots comparing the Cretan populations with the population of Armenia.

1. The top two principal components are shown. The Armenian population is placed close to the Cretanpopulations occupying the center of the island.
2. The top three principal components are shown. There is clear separation between the two populations in
   the third principal component.

**SUPPLEMENTARY FIGURE 24**


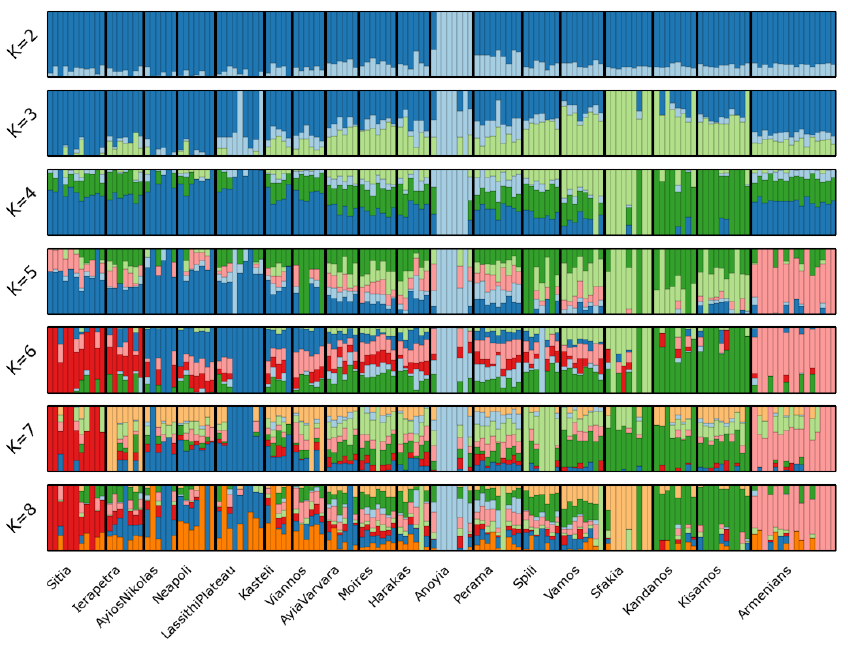


**Supplementary Figure 24.** ADMIXTURE plot of Cretans and Armenians. At values of K larger than five, there is clear separation between the Cretan populations and the Armenian population. Limited Armenian ancestry seems to exist mostly in the central Cretan populations for larger values of K.

**SUPPLEMENTARY FIGURE 25**


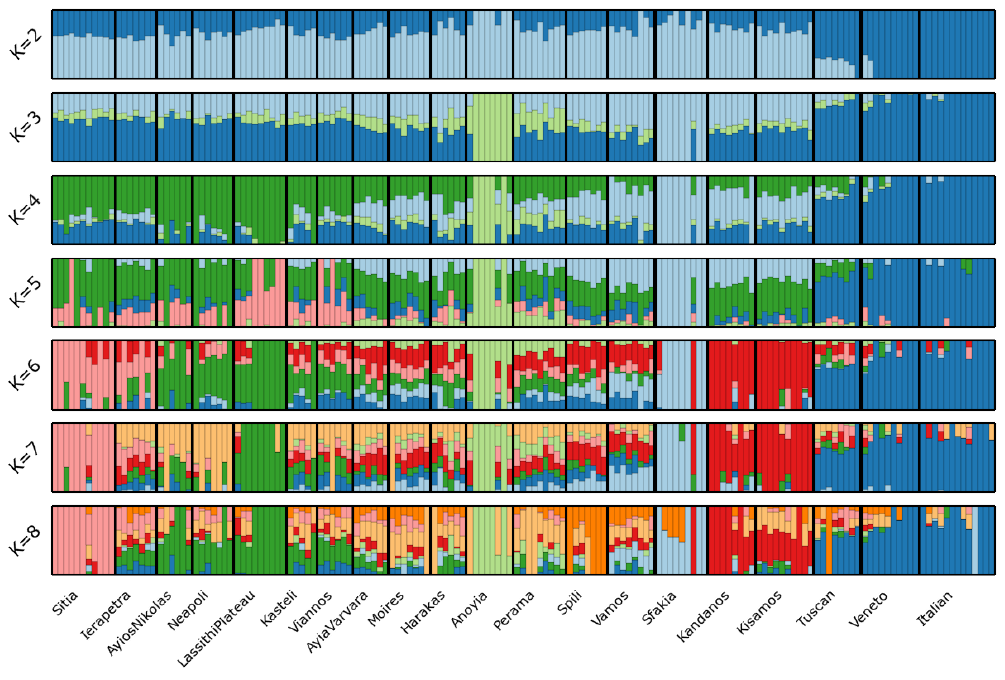


**Supplementary Figure 25.** ADMIXTURE analysis results for the Cretans, Venetians, and two Italian populations. A range of two to eight hypothetical ancestral populations (K) is used. For all values of K, a small amount of shared ancestry appears between the Cretans and the Italian populations. Some gene flow can be observed for values of K larger than four. For values of K larger than five, this genetic flow becomes minimal in the subpopulations of Sfakia, Lassithi, and Anoyia and becomes more concentrated in the central populations.

**Supplementary Table 1. The 17 Cretan subpopulations included in the study.** Number of samples before and after QC.

| Name | District | Original Samples | Samples After QC |
| --- | --- | --- | --- |
| Sitia | Lassithi | 11 | 11 |
| Ierapetra | Lassithi | 7 | 7 |
| AyiosNikolas | Lassithi | 6 | 6 |
| Neapoli | Lassithi | 7 | 7 |
| LassithiPlateau | Lassithi | 10 | 9 |
| Kasteli | Heraclion | 6 | 5 |
| Viannos | Heraclion | 6 | 6 |
| AyiaVarvara | Heraclion | 6 | 6 |
| Moires | Heraclion | 7 | 7 |
| Harakas | Heraclion | 6 | 6 |
| Anoyia | Rethymnon | 8 | 8 |
| Perama | Rethymnon | 9 | 9 |
| Spili | Rethymnon | 7 | 7 |
| Vamos | Chania | 8 | 8 |
| Sfakia | Chania | 10 | 9 |
| Kandanos | Chania | 10 | 8 |
| Kisamos | Chania | 10 | 10 |
| Total | | 134 | 129 |

**Supplementary Table 2. Populations used in this study.** Sample sizes are after QC. For all IBD analyses, the following populations were combined: Russian [6], Russia_A [3] and Russians [4] into Russia; Adygei [3] and Adygei [6] into Adygei; Belarussian [1] and Belorussians [2] into Belarussian.

| Population | Region | Samples | Reference | Chip |
| --- | --- | --- | --- | --- |
| **Belarussianª** | East Europe | 8 | 1 | IlluminaHuman660WQuad |
| **Belorussiansª** | East Europe | 9 | 2 | IlluminaHuman610Quad |
| **Chuvashª** | East Europe | 41 | 3 | IlluminaHuman650Y |
| **Marisª** | East Europe | 15 | 4 | IlluminaHuman610Quad |
| **Mordoviansª** | East Europe | 15 | 5 | IlluminaHuman610Quad |
| **Russia_Aª** | East Europe | 21 | 3 | IlluminaHuman650Y |
| **Russianª** | East Europe | 25 | 6 | IlluminaHuman650Y |
| **Russiansª** | East Europe | 1 | 4 | IlluminaHuman610Quad |
| **Ukraniansª** | East Europe | 20 | 5 | IlluminaHuman610Quad |
| **Estoniansª** | North Europe | 15 | 4 | IlluminaHumanHap650Yv3 |
| **Finnsª** | North Europe | 25 | 3 | IlluminaHuman650Y |
| **Lithuaniansª** | North Europe | 10 | 2 | IlluminaHuman610Quad |
| **Swedishª** | North Europe | 18 | 7 | HumanOmniExpress |
| **Germanª** | Central Europe | 13 | 8 | IlluminaHuman660WQuad |
| **Hungariansª** | Central Europe | 41 | 3 | IlluminaHuman650Y |
| **Polishª** | Central Europe | 17 | 7 | HumanOmniExpress |
| **Romaniansª** | Central Europe | 16 | 2 | IlluminaHuman610Quad |
| **Danesª** | West Europe | 43 | 3 | IlluminaHuman650Y |
| **Frenchª** | West Europe | 29 | 6 | IlluminaHuman650Y |
| **Irishª** | West Europe | 19 | 3 | IlluminaHuman650Y |
| **Orcadianª** | West Europe | 16 | 6 | IlluminaHuman650Y |
| **Basqueª** | SW Europe | 24 | 6 | IlluminaHuman650Y |
| **IBSª** | SW Europe | 107 | 9 | IlluminaOmni2.5M |
| **Spaniardsª** | SW Europe | 12 | 2 | IlluminaHuman610Quad |
| **Andalusiaª** | SW Europe | 9 | present | IlluminaOmni2.5M |
| **Italianª** | South Europe | 13 | 6 | IlluminaHuman650Y |
| **Sardinianª** | South Europe | 28 | 6 | IlluminaHuman650Y |
| **Tuscanª** | South Europe | 8 | 6 | IlluminaHuman650Y |
| **Sicilyª** | South Europe | 20 | present | IlluminaOmni2.5M |
| **TSI** | South Europe | 102 | 9 | IlluminaOmni2.5M |
| **Venetoª** | South Europe | 10 | present | IlluminaOmni2.5M |
| **Bulgariansª** | SE Europe | 13 | 5 | IlluminaHuman610Quad |
| **Creteª** | SE Europe | 129 | present | IlluminaOmni2.5M |
| **Cyprusª** | SE Europe | 30 | present | IlluminaOmni2.5M |
| **Dodecaneseª** | SE Europe | 10 | present | IlluminaOmni2.5M |
| **Peloponneseª** | SE Europe | 229 | present | IlluminaOmni2.5M |
| **Serbiaª** | SE Europe | 20 | present | IlluminaOmni2.5M |
| **Ashkenazi** | Europe | 22 | 3 | IlluminaHuman650Y |
| **CEU** | Europe | 174 | 9 | IlluminaOmni2.5M |
| **Abhkasiansª** | Caucasus | 20 | 5 | IlluminaHuman610Quad |
| **Adygeiª** | Caucasus | 21 | 3 | IlluminaHuman650Y |
| **Adygeiª** | Caucasus | 17 | 6 | IlluminaHuman650Y |
| **Armeniansª** | Caucasus | 16 | 5 | IlluminaHuman610Quad |
| **Chechensª** | Caucasus | 20 | 5 | IlluminaHuman610Quad |
| **Georgianª** | Caucasus | 10 | 7 | HumanOmniExpress |
| **Georgiansª** | Caucasus | 20 | 2 | IlluminaHuman610Quad |
| **Kumyksª** | Caucasus | 14 | 5 | IlluminaHuman610Quad |
| **Altaiansª** | Siberia | 3 | 4 | IlluminaHuman660WQuad |
| **Dolgansª** | Siberia | 1 | 4 | IlluminaHuman660WQuad |
| **Evensª** | Siberia | 2 | 4 | IlluminaHuman660WQuad |
| **Selkupsª** | Siberia | 7 | 4 | IlluminaHuman660WQuad |
| **Yakutª** | Siberia | 21 | 3 | IlluminaHuman650Y |
| **Kalashª** | Hindu-Kush | 25 | 6 | IlluminaHuman650Y |
| **Iraniansª** | Middle East | 18 | 2 | IlluminaHuman610Quad |
| **Kazakhsª** | Middle East | 18 | 4 | IlluminaHuman660WQuad |
| **Kyrgyziansª** | Middle East | 19 | 4 | IlluminaHuman660WQuad |
| **Turkmensª** | Middle East | 15 | 5 | IlluminaHuman610Quad |
| **Uzbeksª** | Middle East | 4 | 4 | IlluminaHuman660WQuad |
| **Druzeª** | Near East | 47 | 6 | IlluminaHuman650Y |
| **Jordaniansª** | Near East | 19 | 2 | IlluminaHuman660WQuad |
| **Lebaneseª** | Near East | 7 | 2 | IlluminaHuman610Quad |
| **Palestinianª** | Near East | 51 | 6 | IlluminaHuman650Y |
| **Samaritansª** | Near East | 38 | 3 | IlluminaHuman650Y |
| **Syriansª** | Near East | 12 | 2 | IlluminaHuman610Quad |
| **Kurds** | Near East | 6 | 5 | IlluminaHuman610Quad |
| **Bedouinª** | Arabia | 48 | 6 | IlluminaHuman650Y |
| **Saudisª** | Arabia | 19 | 2 | IlluminaHuman610Quad |
| **Yemeneseª** | Arabia | 5 | 2 | IlluminaHuman610Quad |
| **Yemenitesª** | Arabia | 37 | 3 | IlluminaHuman650Y |
| **Ethiopiansª** | East Africa | 31 | 3 | IlluminaHuman650Y |
| **Mozabiteª** | East Africa | 30 | 6 | IlluminaHuman650Y |
| **Algeria** | North Africa | 19 | 10 | Affymetrix 6.0 |
| **Egypt** | North Africa | 19 | 10 | Affymetrix 6.0 |
| **Libya** | North Africa | 17 | 10 | Affymetrix 6.0 |
| **Morocco_N** | North Africa | 18 | 10 | Affymetrix 6.0 |
| **Morocco_S** | North Africa | 16 | 10 | Affymetrix 6.0 |
| **Sahara_Occ** | North Africa | 18 | 10 | Affymetrix 6.0 |
| **Tunisia** | North Africa | 18 | 10 | Affymetrix 6.0 |

ª Population was included in the Refined IBD analysis.

**Supplementary Table 3. Populations sharing IBD with West Crete.** All values are based on identity by descent (IBD) segments in cross-population pairs, where a cross-population pair is a pair of individuals such that one individual is from West Crete (n=42) and the other is from the listed population. “Proportion of Pairs with IBD” is the proportion of possible cross-population pairs that share at least one 2 cM IBD segment. A higher proportion indicates that more individuals in the two populations share some IBD with each other. “Mean (SD) Length” is the average and standard deviation of the segment lengths. Longer segments tend to have been inherited from more recent common ancestors. “Mean Pairwise IBD (cM)” is the average amount of IBD shared between an individual in West Crete and an individual in the partner population, calculated as total cross-population IBD divided by the number of possible cross-population pairs.

| Population | N | Proportion of Pairs With IBD | Mean (SD) Segment Length (cM) | Mean Pairwise IBD (cM) |
| --- | --- | --- | --- | --- |
| Andalusia | 9 | 14.0% | 2.44 (0.40) | 0.37 |
| Armenia | 16 | 7.6% | 2.53 (0.72) | 0.20 |
| Basque | 24 | 12.9% | 2.48 (0.69) | 0.34 |
| Bulgaria | 13 | 28.0% | 2.62 (0.70) | 0.89 |
| Caucasus^a^ | 122 | 11.2% | 2.58 (0.68) | 0.31 |
| Central Crete | 36 | 66.5% | 3.46 (1.68) | 5.37 |
| Chuvash | 41 | 30.1% | 2.57 (0.63) | 0.96 |
| East Crete | 51 | 45.4% | 3.31 (1.49) | 2.21 |
| France | 29 | 17.2% | 2.55 (0.79) | 0.49 |
| Hungary | 41 | 30.1% | 2.66 (0.77) | 0.97 |
| Iberia | 107 | 13.2% | 2.59 (0.87) | 0.37 |
| Ireland | 22 | 19.9% | 2.51 (0.78) | 0.55 |
| Italy | 13 | 16.3% | 2.50 (0.66) | 0.43 |
| Middle East^b^ | 74 | 8.9% | 2.62 (0.97) | 0.25 |
| Near East^c^ | 222 | 7.2% | 2.62 (0.79) | 0.21 |
| Poland | 17 | 42.0% | 2.60 (0.66) | 1.48 |
| Romania | 16 | 26.0% | 2.67 (0.81) | 0.82 |
| Russia | 47 | 36.5% | 2.55 (0.64) | 1.18 |
| Sardinia | 28 | 8.5% | 2.55 (0.79) | 0.23 |
| Serbia | 20 | 35.6% | 2.60 (0.70) | 1.16 |
| Sicily | 20 | 12.6% | 2.48 (0.60) | 0.35 |
| Tuscany | 8 | 13.4% | 2.49 (0.54) | 0.36 |
| Ukraine | 20 | 39.9% | 2.60 (0.61) | 1.37 |
| Veneto | 10 | 17.1% | 2.61 (0.65) | 0.48 |

^a^Caucasus: Abhkasians, Adygei, Chechens, Georgians, Kumyks

^b^Middle East: Iranians, Kazakhs, Kyrgyzians, Turkmens, Uzbeks

^c^Near East: Bedouin, Druze, Jordanians, Lebanese, Palestinians, Samaritans, Syrians

**Supplementary Table 4. Populations sharing IBD with East Crete.** All values are based on identity by descent (IBD) segments in cross-population pairs, where a cross-population pair is a pair of individuals such that one individual is from East Crete (n=51) and the other is from the listed population. “Proportion of Pairs with IBD” is the proportion of possible cross-population pairs that share at least one 2 cM IBD segment. A higher proportion indicates that more individuals in the two populations share some IBD with each other. “Mean (SD) Length” is the average and standard deviation of the segment lengths. Longer segments tend to have been inherited from more recent common ancestors. “Mean Pairwise IBD (cM)” is the average amount of IBD shared between an individual in East Crete and an individual in the partner population, calculated as total crosspopulation IBD divided by the number of possible cross-population pairs.

| Population | N | Proportion of Pairs With IBD | Mean (SD) Segment Length (cM) | Mean Pairwise IBD (cM) |
| --- | --- | --- | --- | --- |
| Andalusia | 9 | 14.2% | 2.53 (0.79) | 0.39 |
| Armenia | 16 | 9.2% | 2.68 (0.81) | 0.26 |
| Basque | 24 | 11.9% | 2.47 (0.58) | 0.32 |
| Bulgaria | 13 | 26.6% | 2.64 (0.81) | 0.82 |
| Caucasus^ª^ | 122 | 10.8% | 2.64 (0.83) | 0.31 |
| Central Crete | 36 | 48.9% | 3.32 (1.53) | 2.63 |
| Chuvash | 41 | 27.8% | 2.61 (0.72) | 0.88 |
| France | 29 | 17.4% | 2.46 (0.66) | 0.49 |
| Hungary | 41 | 28.6% | 2.55 (0.66) | 0.89 |
| Iberia | 107 | 13.2% | 2.48 (0.60) | 0.36 |
| Ireland | 22 | 20.7% | 2.51 (0.69) | 0.57 |
| Italy | 13 | 15.7% | 2.61 (0.81) | 0.45 |
| Middle East^b^ | 74 | 8.7% | 2.61 (0.75) | 0.25 |
| Near East^c^ | 222 | 6.6% | 2.58 (0.78) | 0.18 |
| Poland | 17 | 39.5% | 2.64 (0.79) | 1.29 |
| Romania | 16 | 25.7% | 2.61 (0.71) | 0.85 |
| Russia | 47 | 31.8% | 2.61 (0.68) | 0.98 |
| Sardinia | 28 | 7.8% | 2.55 (0.79) | 0.21 |
| Serbia | 20 | 32.8% | 2.72 (0.90) | 1.06 |
| Sicily | 20 | 12.6% | 2.56 (0.74) | 0.35 |
| Tuscany | 8 | 11.0% | 2.54 (0.81) | 0.29 |
| Ukraine | 20 | 41.6% | 2.65 (0.75) | 1.39 |
| Veneto | 10 | 13.9% | 2.65 (0.85) | 0.42 |
| West Crete | 42 | 45.4% | 3.31 (1.49) | 2.21 |

^a^Caucasus: Abhkasians, Adygei, Chechens, Georgians, Kumyks

^b^Middle East: Iranians, Kazakhs, Kyrgyzians, Turkmens, Uzbeks

^c^Near East: Bedouin, Druze, Jordanians, Lebanese, Palestinians, Samaritans, Syrians

**Supplementary Table 5. LD-based inference of admixture times and proportions for the Cretan population using ALDER.** ALDER was run in 1-reference mode for different reference populations as sources of ancestry for the Cretans. We show reference populations with a clear signal of admixture (Z-score of exponential amplitude amp_exp and of Mixture lower bound ≥ 4). We also show in italics at the bottom of the table some populations of interest without a clear signal of admixture (notice implausibly wide date ranges). Present-day is taken as 1930 CE and generation length of 29 years and a 95% confidence interval (±1.96s.e.) is given.

| **Reference population** | Generations | Std. error | Date (low) | Date (high) | Mixture lower bound | Std. error |
| --- | --- | --- | --- | --- | --- | --- |
| Biaka_Pygmy | 36.5 | 6.8 | 485 | 1258 | 0.4 | 0.1 |
| San | 55.3 | 9.9 | -236 | 889 | 0.4 | 0.1 |
| Mbuti_Pygmy | 58.8 | 9.8 | -332 | 782 | 0.5 | 0.1 |
| YRI | 42 | 5.3 | 411 | 1013 | 0.7 | 0.1 |
| LWK | 44.7 | 6.5 | 264 | 1003 | 0.8 | 0.1 |
| Bantu | 50.7 | 7.5 | 33 | 886 | 0.8 | 0.1 |
| Yoruba | 50.4 | 7 | 71 | 866 | 0.8 | 0.1 |
| Mandenka | 52.7 | 7.5 | -25 | 828 | 0.8 | 0.1 |
| MKK | 48.3 | 6.2 | 177 | 882 | 1.2 | 0.1 |
| Koryak | 54.6 | 12.5 | -364 | 1057 | 1.2 | 0.2 |
| Even | 59.5 | 11.1 | -426 | 835 | 1.3 | 0.2 |
| Dolgan | 46.1 | 10.4 | 2 | 1184 | 1.4 | 0.4 |
| Hezhen | 50.8 | 4.9 | 178 | 735 | 1.4 | 0.3 |
| Miao | 55.4 | 7.3 | -92 | 738 | 1.4 | 0.3 |
| Oroqen | 60.6 | 10 | -396 | 741 | 1.5 | 0.3 |
| JPT | 55.8 | 9.8 | -245 | 869 | 1.5 | 0.3 |
| Cambodian | 50.1 | 8.5 | -6 | 960 | 1.6 | 0.4 |
| CHD | 51.9 | 10.4 | -166 | 1016 | 1.6 | 0.4 |
| Daur | 58.1 | 9.4 | -289 | 779 | 1.8 | 0.4 |
| Tuvinian | 50.4 | 6.5 | 99 | 838 | 2.1 | 0.5 |
| Burmese | 51.2 | 12.5 | -265 | 1156 | 2.3 | 0.6 |
| Yukaghir | 51.7 | 12.1 | -257 | 1118 | 2.4 | 0.5 |
| Ho | 58.6 | 12.6 | -486 | 947 | 2.4 | 0.5 |
| Khasi | 75.1 | 14.9 | -1095 | 599 | 2.5 | 0.6 |
| Ethiopian_Jewish | 49.9 | 8.1 | 22 | 943 | 2.7 | 0.3 |
| Ethiopian | 53.7 | 7 | -25 | 771 | 3.0 | 0.3 |
| Chuvash | 37.2 | 6.1 | 504 | 1198 | 6.6 | 1.3 |
| Mordovian | 39.8 | 7.5 | 350 | 1202 | 17.3 | 3.2 |
| Estonian | 41.2 | 9.5 | 195 | 1275 | 19.6 | 3.3 |
| Russian | 45.6 | 5.6 | 289 | 926 | 20.4 | 2.8 |
| Belarusian | 39.2 | 5.8 | 464 | 1123 | 21.4 | 2.8 |
| Ukranian | 39.5 | 5.2 | 489 | 1080 | 27.9 | 2.8 |
| CEU | 38.9 | 5.8 | 472 | 1132 | 28.1 | 3.5 |
| *Armenian* | *43.4* | *21.9* | *-573* | *1916* | *37.2* | *13.1* |
| *Tuscan* | *32.8* | *15.5* | *98* | *1860* | *53.4* | *18.3* |
| *Italian_Bergamo* | *56.9* | *34.7* | *-1692* | *2252* | *45* | *17.7* |
| *Sardinian* | *59.4* | *36.9* | *-1890* | *2305* | *9.2* | *5.6* |
| *French* | *51.0* | *14.6* | *-379* | *1281* | *34.6* | *8* |
| *Druze* | *136.6* | *68.5* | *-5925* | *1862* | *18.8* | *15.8* |
| *Orcadian* | *47.0* | *13.3* | *-189* | *1323* | *16.2* | *4.8* |

Supplementary Table 6. Amount of Cretan ancestry captured by the Andalusians, the Basques and the French. The Andalusians have the highest median value of 7%. The amount of Cretan ancestry that is captured by the Andalusians after removing the “European” influence (as captured by the French) is quite low.

| Reference population | K=4 | K=5 | K=6 | K=7 | K=8 | Median |
| --- | --- | --- | --- | --- | --- | --- |
| Basque | 0.7% | 0.2% | 0.7% | 0.2% | 0.1% | 0.2% |
| Andalusia | 14.4% | 7% | 13% | 3.8% | 1.6% | 7% |
| French | 3% | 1.4% | 5.9% | 1.1% | 0.7% | 1.4% |
| Andalusia minus French | 6.2% | 3.1% | 4% | 1.9% | 1% | 3.1% |

Supplementary Table 7. Amount of Cretan ancestry captured by Near Eastern populations. This amount is quite limited (median values of 1.2% for the Jordanians, 2.9% for the Lebanese, and 6.5% for the Syrians). The only exception is the Kurdish population, for small values of K (K=4 and K=5). Larger values of K result in considerably smaller values of shared ancestry between the Kurds and the Cretans, with a median of 4.5%.

| Reference population | K=4 | K=5 | K=6 | K=7 | K=8 | Median |
| --- | --- | --- | --- | --- | --- | --- |
| Kurds | 19.1% | 21% | 1.4% | 1.8% | 4.5% | 4.5% |
| Jordanians | 1.2% | 1.2% | 1.6% | 0.9% | 0.7% | 1.2% |
| Lebanese | 2.9% | 3.4% | 3.1% | 0.3% | 1.3% | 2.9% |
| Syrians | 6.6% | 6.5% | 8.3% | 4.7% | 2.6% | 6.5% |

Supplementary Table 8. Amount of Cretan ancestry captured by the population of Veneto. The median value is 5.2%; after subtracting the Italian ancestry from the Veneto population, the Cretan ancestry that is captured by the residual vector representing the Veneto population minus the Italian ancestry is practically zero.

| Reference population | K=4 | K=5 | K=6 | K=7 | K=8 | Median |
| --- | --- | --- | --- | --- | --- | --- |
| Veneto | 13.8% | 5.3% | 5.2% | 4% | 2.5% | 5.2% |
| Italian | 12.8% | 4.2% | 4.3% | 3.60% | 2% | 4.2% |
| Veneto – Italian | 0.1% | 0.1% | 0.1% | 0.1% | 0.1% | 0.1% |

**Supplementary Table 9. Populations sharing IBD with Sicily.** All values are based on identity by descent (IBD) segments in cross-population pairs, where a cross-population pair is a pair of individuals such that one individual is from Sicily (n=20) and the other is from the listed population. “Proportion of Pairs with IBD” is the proportion of possible cross-population pairs that share at least one 2 cM IBD segment. A higher proportion indicates that more individuals in the two populations share some IBD with each other. “Mean (SD) Length” is the average and standard deviation of the segment lengths. Longer segments tend to have been inherited from more recent common ancestors. “Mean Pairwise IBD (cM)” is the average amount of IBD shared between an individual in Sicily and an individual in the partner population, calculated as total cross-population IBD divided by the number of possible cross-population pairs.

| Population | N | Proportion of Pairs With IBD | Mean (SD) Segment Length (cM) | Mean Pairwise IBD (cM) |
| --- | --- | --- | --- | --- |
| Andalusia | 9 | 16.7% | 2.49 (0.70) | 0.43 |
| Basque | 24 | 24.2% | 2.56 (0.59) | 0.69 |
| Bulgaria | 13 | 21.2% | 2.63 (0.74) | 0.65 |
| Chuvash | 41 | 20.6% | 2.49 (0.59) | 0.59 |
| Denmark | 43 | 27.9% | 2.52 (0.62) | 0.83 |
| Finland | 25 | 26.0% | 2.54 (0.53) | 0.81 |
| France | 29 | 24.1% | 2.49 (0.58) | 0.69 |
| Hungary | 41 | 22.6% | 2.54 (0.61) | 0.65 |
| Iberia | 107 | 16.8% | 2.51 (0.74) | 0.46 |
| Ireland | 22 | 23.4% | 2.50 (0.44) | 0.68 |
| Italy | 13 | 18.5% | 2.45 (0.49) | 0.48 |
| Russia | 47 | 24.0% | 2.53 (0.62) | 0.71 |
| Sardinia | 28 | 13.6% | 2.68 (0.70) | 0.39 |
| Serbia | 20 | 25.5% | 2.68 (0.85) | 0.79 |
| Tuscany | 8 | 18.1% | 2.32 (0.28) | 0.49 |
| Ukraine | 20 | 26.8% | 2.60 (0.74) | 0.87 |
| Veneto | 10 | 19.5% | 2.41 (0.51) | 0.52 |

**Supplementary Table 10**. IBD shared with Cyprus (n=30); populations are sorted alphabetically. All values are based on IBD segments in cross-population pairs, where a cross-population pair is a pair of individuals such that one individual is from Cyprus and the other is from the listed population. “Proportion of Pairs With IBD” is the proportion of possible cross-population pairs that share at least one 2 cM IBD segment. A higher proportion indicates that more individuals in the two populations share some IBD with each other. “Mean (SD) Length” is the average and standard deviation of the segment lengths. Longer segments tend to have been inherited from more recent common ancestors. “Mean Pairwise IBD (cM)” is the average amount of IBD shared between an individual in Cyprus and an individual in the partner population, calculated as total cross-population IBD divided by the number of possible cross-population pairs.

| Population | N | Proportion of Pairs With IBD | Mean (SD) Segment Length (cM) | Mean Pairwise IBD (cM) |
| --- | --- | --- | --- | --- |
| Andalusia | 9 | 8.9% | 2.47 (0.55) | 0.23 |
| Basque | 24 | 9.3% | 2.68 (0.90) | 0.26 |
| Bulgaria | 13 | 13.1% | 2.53 (0.55) | 0.36 |
| Chuvash | 41 | 13.4% | 2.62 (0.69) | 0.39 |
| Denmark | 43 | 10.5% | 2.46 (0.56) | 0.28 |
| Finland | 25 | 15.5% | 2.47 (0.46) | 0.41 |
| France | 29 | 10.3% | 2.45 (0.61) | 0.26 |
| Hungary | 41 | 14.5% | 2.58 (0.68) | 0.40 |
| Iberia | 107 | 8.6% | 2.56 (0.94) | 0.23 |
| Ireland | 22 | 10.0% | 2.48 (0.73) | 0.25 |
| Italy | 13 | 5.9% | 2.32 (0.26) | 0.15 |
| Russia | 47 | 15.6% | 2.56 (0.60) | 0.45 |
| Sardinia | 28 | 6.1% | 2.58 (0.83) | 0.17 |
| Serbia | 20 | 17.0% | 2.59 (0.67) | 0.50 |
| Sicily | 20 | 10.2% | 2.61 (0.75) | 0.27 |
| Tuscany | 8 | 7.9% | 2.53 (0.67) | 0.23 |
| Ukraine | 20 | 17.7% | 2.55 (0.86) | 0.50 |
| Veneto | 10 | 6.0% | 2.34 (0.31) | 0.15 |

**Supplementary References**

1. Kushniarevich, A., Utevska, O., Chuhryaeva, M., Agdzhoyan, A., Dibirova, K., Uktveryte, I., Möls, M.,

Mulahasanovic, L., Pshenichnov, A., Frolova, S., et al. (2015). Genetic heritage of the Balto-Slavic

speaking populations: A synthesis of autosomal, mitochondrial, and Y-chromosomal data. PLoS ONE 10, e0135820.

1. Behar, D.M., Yunusbayev, B., Metspalu, M., Metspalu, E., Rosset, S., Parik, J., Rootsi, S., Chaubey, G.,

Kutuev, I., Yudkovsky, G., et al. (2010). The genome-wide structure of the Jewish people. Nature 466, 238-242.

1. Rajeevan, H., Soundararajan, U., Kidd, J.R., Pakstis, A.J., and Kidd, K.K. (2011). ALFRED: An allele

frequency resource for research and teaching. Nucleic Acids Res., gkr924.

1. Raghavan, M., Skoglund, P., Graf, K.E., Metspalu, M., Albrechtsen, A., Moltke, I., Rasmussen, S., Stafford,

T.W., Jr., Orlando, L., et al. (2013). Upper Paleolithic Siberian genome reveals dual ancestry of Native

Americans. Nature 505, 87-91.

1. Yunusbayev, B., Metspalu, M., Järve, M., Kutuev, I., Rootsi, S., Metspalu, E., Behar, D.M., Varendi, K.,

Sahakyan, H., Khusainova, R., et al. (2011). The Caucasus as an asymmetric semipermeable barrier to

ancient human migrations. Mol. Biol. Evol. 29, 359-365.

1. Li, J.Z., Absher, D.M., Tang, H., Southwick, A.M., Casto, A.M., Ramachandran, S., Cann, H.M., Barsh,

G.S., Feldman, M., Cavalli-Sforza, L.L., et al. (2008). Worldwide human relationships inferred from

genome-wide patterns of variation. Science 319, 1100-1104.

1. Behar, D.M., Metspalu, M., Baran, Y., Kopelman, N.M., Yunusbayen, B., Gladstein, A., Tzur, S.,

Sahakyan, H., Bahmanimehr, A., Yepiskoposyan, L., et al. (2013). No evidence from genome-wide data of a Khazar origin for the Ashkenazi Jews. Hum. Biol. 85, 859-900.

1. Yunusbayev, B., Metspalu, M., Valeev, A., Litvinov, S., Valiev, R., Akhmetova, V., Balanovska, E.,

Balanovsky, O., Turdikulova, S., et al. (2015). The genetic legacy of the expansion of Turkic-speaking

nomads across Eurasia. PLoS Genet. 11, e1005068.

1. Auton, A., Brooks, L.D., Durbin, R.M., Garrison, E.P., Kang, H.M., Korbel, J.O., Marchini, J.L., McCarthy,

S., McVean, G.A., and Abecasis, G.R.; 1000 Genomes Project Consortium (2015). A global reference for

human genetic variation. Nature 526, 68-74.

1. Henn, B.M., Botigué, L.R., Gravel, S., Wang, W., Brisbin, A., Byrnes, J.K., Fadhlaoui-Zid, K., Zalloua, P.A., Moreno-Estrada, A., Bertranpetit, J., et al. (2012). Genomic ancestry of North Africans supports back to-Africa migrations. PLoS Genet. 8, e1002397.
2. Stamatoyannopoulos, G., Bose, A., Teodosiadis, A., Tsetsos, F., Plantinga, A., Psatha, N., Zogas, N., Yannaki, E., Zalloua, P., Kidd, K.K., Browning, B.L., Stamatoyannopoulos, J.A., Paschou, P., Drineas, P. (2017). Genetics of the Peleponnesean Populations and the Theory of Extinction of the Medieval Peloponnesean Greeks, European Journal of Human Genetics (EJHG), 25(5): 637-645.
